# High-fat diet activates splenic NOD1 and enhances neutrophil recruitment and neutrophil extracellular traps release in the spleen of ApoE-deficient mice

**DOI:** 10.1007/s00018-022-04415-x

**Published:** 2022-07-05

**Authors:** Victoria Fernández-García, Silvia González-Ramos, José Avendaño-Ortiz, Paloma Martín-Sanz, Diego Gómez-Coronado, Carmen Delgado, Antonio Castrillo, Lisardo Boscá

**Affiliations:** 1 Instituto de Investigaciones Biomédicas Alberto Sols (CSIC-UAM), Arturo Duperier 4, 28029 Madrid, Spain; 2 Centro de Investigación Biomédica en Red en Enfermedades Cardiovasculares (CIBERCV), Monforte de Lemos 3-5, 28029 Madrid, Spain; 3 Instituto de Investigación Sanitaria del Hospital Universitario La Paz, IdiPAZ., C. de Pedro Rico, 6, 28029 Madrid, Spain; 4 Centro de Investigación Biomédica en Red de Enfermedades Hepáticas y Digestivas (CIBERehd), Monforte de Lemos 3-5, 28029 Madrid, Spain; 5 Servicio de Bioquímica-Investigación, Hospital Universitario Ramón y Cajal, Ctra. M-607 9,100, 28034 Madrid, Spain; 6 Centro de Investigación Biomédica en Red de Fisiopatología de la Obesidad y Nutrición (CIBERobn), Monforte de Lemos 3-5, 28029 Madrid, Spain; 7 Unidad de Biomedicina (Unidad Asociada Al CSIC), Instituto Universitario de Investigaciones Biomédicas y Sanitarias (IUIBS) de la Universidad de Las Palmas de Gran Canaria, Las Palmas, Spain

**Keywords:** Spleen, NETosis, Hypercholesterolemia, Atherogenesis, Partial splenectomy

## Abstract

In the course of atherogenesis, the spleen plays an important role in the regulation of extramedullary hematopoiesis, and in the control of circulating immune cells, which contributes to plaque progression. Here, we have investigated the role of splenic nucleotide-binding oligomerization domain 1 (NOD1) in the recruitment of circulating immune cells, as well as the involvement of this immune organ in extramedullary hematopoiesis in mice fed on a high-fat high-cholesterol diet (HFD). Under HFD conditions, the absence of NOD1 enhances the mobilization of immune cells, mainly neutrophils, from the bone marrow to the blood. To determine the effect of NOD1-dependent mobilization of immune cells under pro-atherogenic conditions, *Apoe*^*−/−*^ and *Apoe*^*−/−*^*Nod1*^*−/−*^ mice fed on HFD for 4 weeks were used. Splenic NOD1 from *Apoe*^*−/−*^ mice was activated after feeding HFD as inferred by the phosphorylation of the NOD1 downstream targets RIPK2 and TAK1. Moreover, this activation was accompanied by the release of neutrophil extracellular traps (NETs), as determined by the increase in the expression of peptidyl arginine deiminase 4, and the identification of citrullinated histone H3 in this organ. This formation of NETs was significantly reduced in *Apoe*^*−/−*^*Nod1*^*−/−*^ mice. Indeed, the presence of Ly6G^+^ cells and the lipidic content in the spleen of mice deficient in *Apoe* and *Nod1* was reduced when compared to the *Apoe*^*−/−*^ counterparts, which suggests that the mobilization and activation of circulating immune cells are altered in the absence of NOD1. Furthermore, confirming previous studies, *Apoe*^*−/−*^*Nod1*^*−/−*^ mice showed a reduced atherogenic disease, and diminished recruitment of neutrophils in the spleen, compared to *Apoe*^*−/−*^ mice. However, splenic artery ligation reduced the atherogenic burden in *Apoe*^*−/−*^ mice an effect that, unexpectedly was lost in *Apoe*^*−/−*^*Nod1*^*−/−*^ mice. Together, these results suggest that neutrophil accumulation and activity in the spleen are driven in part by NOD1 activation in mice fed on HFD, contributing in this way to regulating atherogenic progression.

**Supplementary Information:**

The online version contains supplementary material available at 10.1007/s00018-022-04415-x.

## Introduction

The classical physiological roles of the spleen involve, among others, blood filtration and the regulation, selection and storage of different immune cell populations. Nevertheless, the most important task of this organ under pathological conditions is its hematopoietic activation [1–5]. This is because the spleen harbors numerous highly differentiated anatomical structures and cells that allow specific functions due to its direct connection to the systemic circulation and further links with the nervous and immune systems [6]. Anatomically, the spleen architecture includes heterogeneous populations of stromal, immune, and endothelial cells, organized in domains with specific microcirculation [7–11]. This organ is divided into two main compartments, the red pulp (RP) and the white pulp (WP), which are significantly different in terms of their structure, morphology, vascular organization, cell composition, and physiological functions. The RP is a highly efficient blood filter responsible for removing non-self-material, platelets, and damaged red blood cells and has large amounts of macrophages that are very active for these purposes. In contrast, the main immune responses to blood antigens occur in the WP, where approximately a quarter of the body's lymphocytes reside. This WP surrounds the central arterioles and has different zones: the periarteriolar lymphoid sheath, the follicles, and the marginal zone. WP also has macrophages but is enriched in other immune cells, such as CD4 and CD8 T cells and B cells, dendritic cells and plasma cells. In addition, some particular immune functions have been ascribed to this versatile organ, such as removal of non-opsonized bacteria, blood cells clearance, and sensing the presence of circulating pathogen-associated molecular patterns (PAMPS). In this regard, splenic nucleotide-binding oligomerization domain 1 (NOD1) and NOD2, synergizing with TLR4 activation, have been involved in the mobilization of hematopoietic stem cells from the bone marrow to the spleen, contributing to the host defense against several pathogens [4, 7, 12–14]. Moreover, leukocyte lineages derived from hematopoietic cells, mainly neutrophils releasing neutrophil extracellular traps (NETs) and macrophages, cells that in turn express NOD molecules, exert key roles in the mobilization of hematopoietic cells under pro-inflammatory conditions. These NETs have been shown to play an essential role in the development of atherosclerosis [15]. In this sense, NETs are capable of triggering the activation of antigen-presenting cells, endothelial cells and platelets, favoring a pro-inflammatory immune scenario. Because both NETs and NET-related cell death or NETosis are considered a fundamental connection between innate immunity, inflammation, oxidative stress and cardiovascular diseases, this work aimed to study these mechanisms under the onset of NOD1 signaling. In fact, NOD1 is related to NET induction [16].

Furthermore, the spleen participates in the modulation of lipid metabolism and plasma lipids content, mechanisms relevant to the onset of atherosclerotic complications and cardiovascular diseases [10, 17–20]. Elevated plasma LDL-cholesterol levels have been observed both in humans and animal models after splenectomy, suggesting a preeminent role for the spleen in LDL catabolism [21–23]. Despite these roles of the spleen in essential immune and metabolic processes, controversies exist regarding the consequences of splenic loss of function. This can occur after a partial or total spleen intervention (i.e.; after ligation of splenic arteries in animal models, traumatic splenectomy, or surgical removal in some splenic pathologies) [8, 22–28]. In contrast, the gain of splenic function has been evidenced in splenomegaly patients [17, 24, 29, 30].

## Materials and methods

### Animal procedures

C57BL/6 (*WT*) and *Apoe*^*−/−*^ mice were obtained from Charles River (JAX mice stock #000664 and #002052, respectively. Barcelona, Spain). Double-knockout *Apoe*^*−/−*^*Nod1*^*−/−*^ mice were generated by crossing *Apoe*^*−/−*^ mice with *Nod1*^*−/−*^ mice as previously described [31, 32]. Only male mice were used for the experiments because these animals are more prone to develop atherogenesis than other genotypes, as previously described [32, 33]. For the spleen surgeries, 8-week-old mice were randomly assigned to either splenic artery ligation or the control group (sham). Mice were intubated and anesthetized with 2% isoflurane. The fur over the left side of the abdomen was carefully shaved, mice were accommodated on a heating pad (37 °C) to avoid temperature loss during the operation and skin was disinfected with betadine and alcohol before the intervention. The spleen was identified and it was ligated with a 7–0 nylon suture around the splenic arteries to mimic a partial splenic loss of function (splenic hilar ligation group). The small incision in the abdomen and the skin was closed by employing absorbable 5–0 sutures and special glue specific for animal tissue (3 M™ Vetbond™ Tissue Adhesive, Saint Paul, MN, USA). Ibuprofen (Dalsy, Mylan, Dublin, Ireland) as analgesic was supplied in drinking water (3 ml of Dalsy per 250 ml of water) and continued for three days after the procedure. The wound healing was monitored daily and adequate recovery after the surgical process was ensured. Three days after sham operation or ligation of splenic arteries, mice were kept on chow or a high-fat diet (HFD, 10.2% hydrogenated coconut oil, 0.75% cholesterol; Ssniff, Soest, Germany) for 4 weeks. After this feeding period, mice were anesthetized intraperitoneally under general anesthesia (ketamine/xylazine combination at 80 mg/kg and 10 mg/kg body weight, respectively) before euthanasia by CO_2_ inhalation. Whole blood was extracted *postmortem* by cardiac puncture and plasma was obtained by centrifugation at 2,000* g* for 10 min at 4ºC.

### Murine neutrophils isolation

Mouse bone marrow-derived neutrophils were isolated from tibias and femurs from *Apoe*^*−/−*^, iE-DAP-treated *Apoe*^*−/−*^ and *Apoe*^*−/−*^* Nod1*^*−/−*^ mice by negative selection using the Neutrophil Isolation Kit (Miltenyi; ref. 130-097-658) and following the manufacturer’s instructions, as previously described [34]. iE-DAP-treated *Apoe*^*−/−*^ mice were challenged intraperitoneally with 1 mg/kg body weight of iE-DAP 24 h before they were sacrificed and after the 4 weeks of HFD to which all the animals were subjected.

### NETs release assays

After murine neutrophil isolation from the bone marrow (femur and tibia), the cells underwent neutrophil extracellular traps (NETs) quantification experiments, following a previous isolation and assay protocol [34]. To this aim, neutrophils (2 × 10^6^) were incubated for 4 h in HBSS (Thermo Fisher) supplemented with 5 mM HEPES; pH 7.4 (Merck). After washing, the cells were resuspended and incubated for 30 min in DMEM containing 10 U/ml of AluI (New England BioLabs, Ipswich, MA. USA). Supernatants with NETs fragments were collected and centrifuged (5 min, 300* g*) to remove the remaining cell debris. Quant-iT PicoGreen dsDNA Assay Kit (Thermo Fisher) was used to measure DNA concentrations according to the manufacturer’s instructions.

### Flow cytometry assays

Mice blood, bone marrow (BM) and spleen samples were used for the flow cytometry assays after keeping the different mice groups under chow or HFD for 4 weeks, as previously described [31, 32]. Briefly, to analyze myeloid cell populations in these tissues, cell suspensions were obtained after centrifugation and lysis (blood) or flushing (BM and spleen) and they were prepared by passing the resulting solutions through a 70 mm cell strainer. After 400* g* centrifugation for 5 min at 4 °C, the pellet was resuspended in HBSS (Thermo Fisher) supplemented with 10 mM HEPES and 0.5% bovine serum albumin (pH 7.4) and incubated for 30 min at 4 °C with: rat APC-Cy7-conjugated mAb against CD45 (1:200; BioLegend, San Diego, CA, USA), rat PE-conjugated mAb against CD115 (1:100; Thermo Fisher), rat PerCpCy5.5- conjugated mAb against Ly6G (1:100; BioLegend), rat FITC-conjugated mAb against Ly6C (1:100; BioLegend), rat APC-conjugated mAb against F4/80 (1:100; BioLegend), rat PECy7-conjugated mAb against Cd11b (1:100; eBioscience), rat PECy7-conjugated mAb against Ly6C (1:100; eBioscience), rat FITC-conjugated mAb against CD4 (1:100; BioLegend), rat APC-conjugated mAb against CD8 (1:100; BioLegend), rat PerCp-conjugated mAb against B220 (1:100; BioLegend). For cell counting, DAPI and absolute counting beads were used (Count-Bright; Thermo Fisher). Flow cytometry was conducted in a FACSCanto II (Becton Dickinson), and leukocyte subsets were defined using FlowJo software (Treestar, Ashland, OR, USA): leukocytes (CD45^+^), neutrophils (CD45^+^ CD11b^+^ Ly6G^+^), inflammatory monocytes (CD45^+^ CD115^+^ CD11b^+^ Ly6C^+^), tissue macrophages (CD45^+^ F4/80^+^), CD4 lymphocytes (CD45^+^ CD4^+^), CD8 lymphocytes (CD45^+^ CD8^+^) and B lymphocytes (CD45^+^ B220^+^).

### Plasma chemoattractants measurement

Eight-week-old mice were fed for 4 weeks with HFD and inflammatory mediators and chemo-attractants (CCL2, CCL5, CXCL1, CXCL2) were quantified in plasma using the Milliplex Map Mouse Cytokine/Chemokine Magnetic Bead Panel (Merck Millipore) in a Luminex (Austin, TX, USA) 100 IS system as per the manufacturer’s specifications.

### Plasma lipidic profile

Four-week HFD-fed mice plasma TAG (triacylglycerides), LDL (low-density lipoprotein cholesterol), HDL (high-density lipoprotein cholesterol), FCHO (free cholesterol), TCHO (total cholesterol), pancreatic lipase and NEFA (non-esterified fatty acids) were determined enzymatically using kinetic colorimetric kits (Spinreact, St Esteve de Bas, Girona, Spain) according to manufacturer’s instructions.

### Histological analysis and lesion quantification

Cryocut cross Sects. (5 µm) of aortic roots were evaluated for conventional hematoxylin–eosin and Oil Red O staining as previously described [31, 32]. Images were captured with a Zeiss Axiophot microscope with a Plan-NEOFLUAR 10x/0.3 objective (Zeiss, Oberkochen, Germany) and a DP70 camera (Olympus, Southend-on-Sea, UK). Atherosclerotic lesion areas in mice hearts and valves, expressed in percentage and mice splenic lipid content in the red pulps and white pulps were obtained as previously described [31, 32]. Briefly, after mouse cardiac perfusion with PBS supplemented with 5 mM of EDTA, mouse hearts were harvested and fixed in 4% paraformaldehyde for 24 h at 4 °C, passed through sucrose gradients at 10% and 20% (PBS supplemented with the respective concentration of sucrose), incubated 24 h in 30% sucrose, embedded in optimal cutting temperature and cryopreserved at − 80 °C. Cryocut cross sections (8 μm) were evaluated for conventional hematoxylin–eosin (HE) staining. Images were captured with a Zeiss Axiophot microscope with a Plan-Neofluar 310/0.3 objective (Carl Zeiss, Oberkochen, Germany) and a DP70 camera (Olympus, Tokyo, Japan). To avoid specific biases due to potential differences in lesion shape, cross sections of the entire lesion were analyzed and averaged. For splenic samples, the same fixation and cryopreservation were performed. After being cut, tissues underwent Oil Red O (Sigma) staining to detect neutral lipids. Both the planimetric area of atherosclerotic plaques (hearts) and the lipids area (spleens) were measured in pixels using ImageJ (NIH) and quantified.

### Immunostaining

Immunofluorescence assays were performed as described before [31, 32]. In brief, mice spleens and hearts were fixed overnight for 24 h at 4 °C, passed through sucrose gradients at 10% and 20% (PBS supplemented with the respective concentration of sucrose), incubated 24 h in 30% sucrose, embedded in optimal cutting temperature and cryopreserved at -80 °C. Afterward, they were sectioned into 5 μm sections with a microtome (Jung RM2055; Leica Microsystems, Wetzlar, Germany). Cryo-section samples slides were rehydrated, subjected to antigen retrieval in 10 mM citrate buffer (pH 6.0), blocked and stained with antibodies specific for mouse Ly6G (1:100; Becton Dickinson), histone 3 citrullinated (1:200; Abcam), CXCL12 (1:100; Abcam), followed by secondary staining using standard procedures. Secondary antibodies for immunofluorescence were Alexa Fluor 647-conjugated anti-rabbit (Thermo Fisher), Alexa Fluor 594-conjugated anti-rat (Thermo Fisher) and FITC-conjugated anti-rat (Sigma). Nuclei were counterstained with DAPI (Thermo Fisher). Immunofluorescence staining of cryo-sections was evaluated in Prolong Gold Antifade mounting medium (Thermo Fisher). Primary control panel was performed with an appropriate isotype control IgG, and secondary controls incubations were performed in the absence of the primary antibody. For the TUNEL assays, the cryo-sections were processed following the manufacturer’s specifications (In Situ Cell Death Detection Kit, Fluorescein; Roche). An LSM710 confocal microscope with a Plan-Apochromat 325/0.8 oil immersion objective (Carl Zeiss) was used to capture images from immunofluorescence staining. Additionally, the white pulp (WP) in these experiments was identified by microscopic observation after hematoxylin/eosin staining and was delimitated within the white lines in the figures, which contained the WP and the marginal zone. The red pulp (RP) was located outside the white lines in the figures. Images were analyzed using ImageJ [National Institutes of Health (NIH), Bethesda, MD, USA] and were processed for presentation with Zen2009 (Carl Zeiss) software.

### Western blot analysis

Mouse splenic samples were snap-frozen in liquid nitrogen and stored at − 80 °C. Subsequent processing was carried out according to previous protocols [31, 32]. In summary, protein extracts from mouse tissues were obtained using ice-cold proprietary detergent in 25 mM Bicine, 150 mM NaCl (pH 7.6) (T-PER: Tissue Protein Extraction Reagent; Thermo Fisher) supplemented with phosphatase cocktail and protease inhibitors (Sigma). Proteins were resolved on SDS-PAGE gels and then transferred to nitrocellulose membranes. Proteins were detected using rabbit pAb against NOD1 (1:500; Abcam), rabbit mAb against phospho-RIPK2 (1:1000; Cell Signaling, Danvers, MA, USA), rabbit pAb against phospho-RIPK2 (1:1000; Cell Signaling), rabbit pAb against LOX1 (1:1000; Abcam), rabbit mAb against phospho-p65 (1:1000; Cell Signaling), mouse mAb anti-ABCA1 (1:1000; Abcam), rabbit mAb against p65 (1:1000; Cell Signaling), rabbit pAb against histone 3 citrullinated (1:1000; Abcam), rabbit mAb against phospho-ERK (1:1000; Cell Signaling), rabbit mAb against ERK (1:1000; Cell Signaling), rabbit mAb against phospho-p38 (1:1000; Cell Signaling), rabbit mAb against p38 (1:1000; Cell Signaling), rabbit mAb against phospho-TAK1 (1:1000; Cell Signaling), rabbit mAb against TAK1 (1:1000; Cell Signaling), rabbit mAb against NOS3 1:1000; Abcam), mouse mAb against α-tubulin (1:4000; Sigma) and horseradish peroxidase–conjugated secondary antibodies (Bio-Rad, Hercules, CA, USA). Protein bands were visualized using a Luminata chemiluminescence detection system (Merck Millipore) and an Image-Quant LAS 500 imager (GE Healthcare Life Sciences, Freiburg, Germany) and were quantified using ImageJ. Intensities of protein bands were expressed as a percentage of those of the tubulin.

### qRT-PCR

Total RNA was isolated by homogenization in a TissueLyser LT with QUIAZOL and eluted using MinElute columns (Qiagen; Madrid, Spain). RNA integrity was assessed by RNA Nano Chip (Agilent Technologies; Madrid, Spain). 250 ng of RNA were retro-transcribed using the High-Capacity cDNA Reverse Transcription Kit (Applied Biosystems; Madrid, Spain). SYBR Green assay was conducted in 7900HT Fast Real-Time PCR System equipment for qRT-PCR detection of the indicated genes (Supplemental Table S1). Calculations were obtained from the measurement of technical triplicates of each sample. The relative amount of mRNA was calculated with the comparative 2^−ΔΔCt^ method using mouse *Hprt1* or human *GAPDH*, respectively, as endogenous control transcripts.

### Quantification and statistical analysis

All the values are expressed as means ± SD. GraphPad Prism 6 (GraphPad Software Inc.; San Diego, CA, USA) was employed to perform the statistical analysis. After calculating for normality by D’Agostino–Pearson omnibus test, a non-parametric test (Mann–Whitney *U* test), or a parametric test (unpaired Student’s *t* test with Welch’s correction) was used as the most appropriate in each case. One-way ANOVA followed by Bonferroni’s post hoc tests was used for multiple comparisons. Statistical significance was considered at *P* values < 0.05. Removal of outliers was performed by the ROUT method. Statistical tests and *P* values are indicated for each panel in the corresponding figure legends. The number of individual animals (*n*) for in vivo and ex vivo experiments is provided in each figure. ‬‬‬‬‬‬

## Results

### Deletion of *Nod1* enhances leukocyte blood accumulation under high-fat and hypercholesterolemic diet (HFD), but activation of NOD1 favors increased levels of splenic myeloid cells

**Fig. 1:**
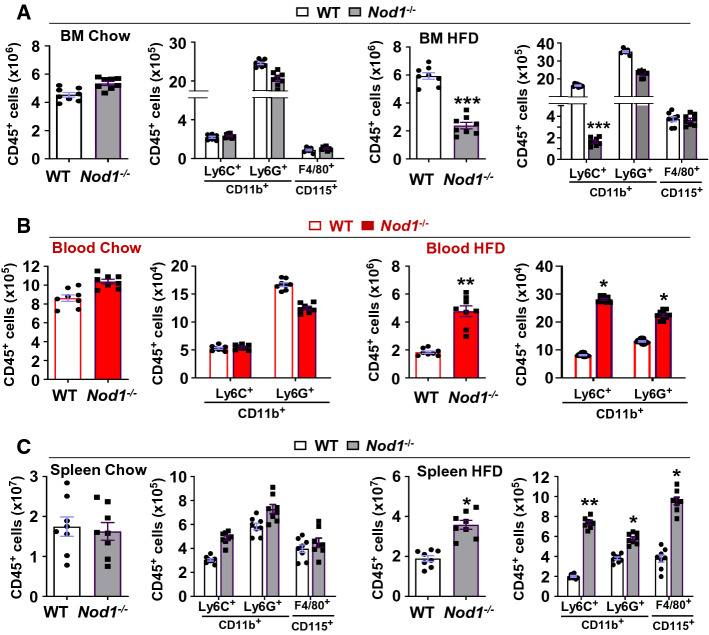
The absence of NOD1 alters bone marrow and circulating immune cells profile in mice fed on a high-fat diet (HFD) for four weeks. Wild type (WT) and *Nod1*^*−/−*^ male mice were fed on a chow or HFD for four weeks and the amount and distribution of CD45^+^ cells were determined in the bone marrow (**A**), blood (**B**) and in the spleen (**C**). The content of CD45^+^ cells and the CD11b^+^Ly6C^+^, CD11b^+^Ly6G^+^ populations were determined. The CD115^+^F4/80^+^ population was quantified in the bone marrow and spleen. Results show the mean ± SD from 8 animals of each condition. Statistical significance was estimated as *p* value calculated by un-paired *t test*; *P < 0.05; **P < 0.01; ***P < 0.005 *vs*. the same nutritional condition in WT mice

First, we determined the effect of genetic deletion of *Nod1* in mice fed on chow or HFD for 4 weeks on the mobilization of immune cells from the bone marrow to the circulation and the spleen (Fig. 1A–C). Representative flow cytometry plots of these populations are provided in Supplementary Figures S1–S3. While there are no significant changes in leukocyte population counts in these three tissues under a chow diet, HFD establishes significant variations between mouse genotypes. These data support the importance of the simultaneous conditions of HFD and NOD1 deficiency to the alterations of the leukocyte populations observed in these three hematopoietic niches: *Nod1*^*−/−*^ HFD-fed mice showed a decrease in bone marrow CD45^+^ cells and an increase in both circulating and splenic levels of these cells, as it is also determined in specific subsets of CD45^+^ cells (Ly6C^+^, Ly6G^+^ and splenic F4/80^+^ cells).

**Fig. 2:**
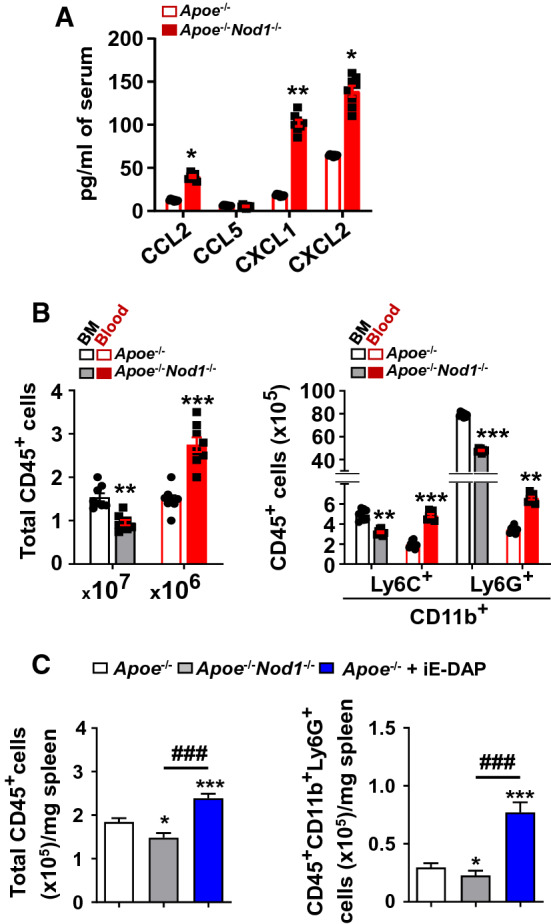
The absence of NOD1 under an *Apoe*^*−/−*^ background enhances the mobilization of CD45^+^ cells from the bone marrow to the blood and their accumulation, while NOD1 activation favors leukocyte ontogeny in the bone marrow and splenic infiltration in mice fed on a high-fat diet (HFD) for four weeks. **A** Circulating of selected chemokines levels in *Apoe*^*−/−*^ and *Apoe*^*−/−*^*Nod1*^*−/−*^ mice after four weeks of HFD. **B** Quantification of bone marrow (BM) and circulating immune cells from mice described in panel A. **C** Analysis of the distribution of CD45^+^ cells and CD11b^+^Ly6G^+^ in the spleen of these mice. To ensure maximal activation of NOD1, *Apoe*^*−/−*^ mice were challenged intraperitoneally with the NOD1-agonist iE-DAP (1 mg/kg body weight) 24 h before sacrifice, and the CD45^+^ and CD11b^+^Ly6G^+^ cells were quantified. Results show the mean ± SD from 9 animals of each condition (*Apoe*^*−/−*^ and *Apoe*^*−/−*^*Nod1*^*−/−*^). Statistical significance was estimated as P value calculated by un-paired *t-test* (panels A, B) or by one-way ANOVA followed by Bonferronis’s post hoc multi-comparisons analysis (panel C); *P < 0.05; **P < 0.01; ***P < 0.005 *vs*. the corresponding *Apoe*^*−/−*^ condition; ^###^P < 0.005 *vs*. the corresponding *Apoe*^*−/−*^*Nod1*^*−/−*^

To better assess the role of splenic NOD1 on leukocyte mobilization under HFD, one of the best models is to study this molecule under an *Apoe*^*−/−*^ background [31, 32], a condition that favors atherogenesis. In this regard, *Apoe*^*−/−*^*Nod1*^*−/−*^ mice fed on a chow diet did not show significant changes in the mobilization of cells from the bone marrow to the blood and spleen (Supplemental Figure S4). However, this situation was changed in *Apoe*^*−/−*^*Nod1*^*−/−*^ mice fed on HFD for 4 weeks. As Fig. 2A shows, serum levels of the chemo-attractants CCL2, CXCL1 (neutrophils) and CXCL2 (monocytes and macrophages) were significantly increased, due to HFD and the fact that, in the absence of NOD1, the cell infiltration in the atheromatous plaque is reduced as previously described [31, 32, 35]. Other chemokines like the chemoattractant CCL5 (mainly a chemotactic factor for T cell recruitment to inflammatory sites) did not exhibit significant changes. These changes agreed with a decrease in the bone marrow CD45^+^ population and an increase in circulating CD45^+^, CD11b^+^Ly6C^+^ and CD11b^+^Ly6G^+^ cells (Fig. 2B and Supplemental Figure S5). Interestingly, *Apoe*^*−/−*^ mice fed on HFD exhibit an increase in CD45^+^ and CD11b^+^Ly6G^+^ cells in the spleen when compared to *Apoe*^*−/−*^*Nod1*^*−/−*^ mice, which suggests that in the absence of NOD1, the infiltration of circulating immune cells is attenuated (Fig. 2C). Moreover, pharmacological activation of NOD1, with the NOD1-agonist iE-DAP, enhances the recruitment of inflammatory cells in the spleen (Fig. 2C and Supplemental Figure S6). This situation has been previously described [32].

In addition to these data, *Apoe*^*−/−*^*Nod1*^*−/−*^ mice exhibited an increase in their circulating leukocytes, mainly due to higher levels of neutrophils and inflammatory monocytes (Supplemental Figure S7A). However, no statistically significant differences were observed between the main circulating cell populations in *Apoe*^*−/−*^* vs*. *Apoe*^*−/−*^*Nod1*^*−/−*^ HFD-fed mice after splenic artery ligation (Supplemental Figure S7B). Moreover, as Supplemental Figure S8A shows, spleen ligation in *Apoe*^*−/−*^ mice resulted in enhanced bone marrow mRNA levels of *Nod1* and in the chemokine *Cxcl12*, which is involved in the chemotaxis of lymphocytes and considered a coronary artery risk factor [3, 36–38]. In addition, *Apoe*^*−/−*^*Nod1*^*−/−*^ sham-operated animals exhibited specific changes in some genes committed to the function of the bone marrow and the differentiation and fate of hematopoietic cells, such as *Spi1, Gcsfr, Kit* and *Cd47* (Supplemental Figure S8B-C). Spleen ligation in *Apoe*^*−/−*^*Nod1*^*−/−*^ mice increased or involved additional specific genes in the bone marrow, such as *Spi1, Gata1, Csfr1, Dngr1* and *Irf8*, but decreased the levels of others, such as *Kit, Gcsfr* and *Cd47*. These data suggest that decreased splenic function alters the normal performance of the bone marrow in *Apoe*^*−/−*^*Nod1*^*−/−*^ mice under HFD (Supplemental Figure S8B). However, *Apoe*^*−/−*^*Nod1*^*−/−*^ mice have minimal differences *vs*. *Apoe*^*−/−*^ counterparts in terms of expression of cell adhesion molecules in the spleen (Supplemental Figure S8C).

### Splenic NOD1 modulates plasma lipid levels and atheroma plaque progression

Since the spleen is a key component in extramedullary hematopoiesis we evaluated the role of NOD1 under pro-atherogenic conditions [1, 4, 18]. As Supplemental Figure S9A-B shows, splenic *Nod1* mRNA levels remained unchanged in mice fed on a chow diet, but were increased in *Apoe*^*−/−*^ mice fed on HFD. *Nod2* mRNA levels did not present important changes under both chow or HFD. Moreover, NOD1 protein levels and activity were increased as deduced by the phosphorylation of the downstream target RIPK2. Furthermore, as previously described [39], the absence of NOD1 in *Apoe*-deficient mice fed on HFD resulted in increased body weight. However, this effect was attenuated after spleen artery ligation, suggesting a role for this organ in the enhancement of body weight. These conditions (sham-operated *vs*. spleen artery ligation) did not alter the spleen mass in mice fed on HFD (Supplemental Figure S9C).

**Fig. 3:**
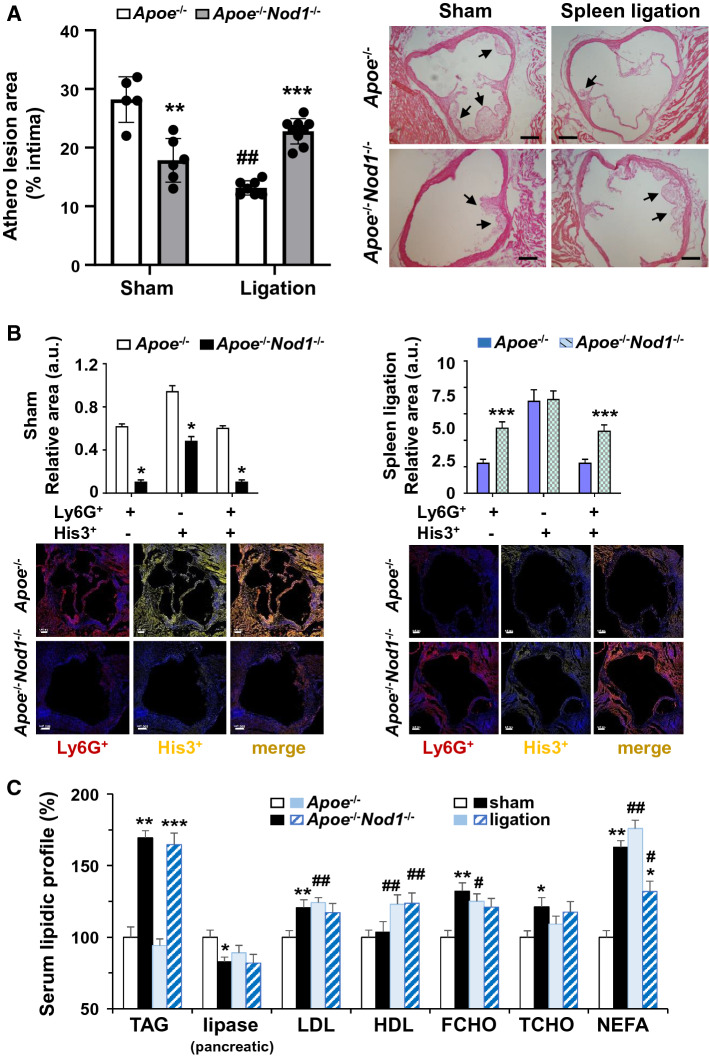
Splenic artery ligation reduces atherogenesis progression in *Apoe*^*−/−*^ mice fed on HFD for four weeks. *Apoe*^*−/−*^ and *Apoe*^*−/−*^*Nod1*^*−/−*^ mice were submitted to sham or splenic artery ligation before starting a 4 weeks HFD. The extent of the atherogenic lesion was determined by histochemistry. **A** Quantification of the atherogenic lesion in *Apoe*^*−/−*^ and *Apoe*^*−/−*^*Nod1*^*−/−*^ mice after different spleen interventions and representative images of the atherogenic lesion in the heart from mice. *Arrows* indicate the presence of atherogenic lesions. **B** Quantification of Ly6G^+^ and citrullinated histone H3 in the atherogenic lesion. **C** Since splenic artery ligation in *Apoe*^*−/−*^ mice delayed the atherogenic progression, blood lipids and pancreatic lipase activity were measured in serum from these animals fed on HFD for four weeks. Values were expressed as percentage *vs*. the sham *Apoe*^*−/−*^ condition. Results show the mean ± SD from 8 animals of each condition (sham and splenic artery ligation of *Apoe*^*−/−*^ and *Apoe*^*−/−*^*Nod1*^*−/−*^ mice). Statistical significance was estimated as P value calculated by un-paired *t* test (panel A) or by one-way ANOVA followed by Bonferronis’s post hoc multi-comparisons analysis (panel B); *P < 0.05; **P < 0.01; ***P < 0.005 *vs*. the corresponding splenic intervention condition (*Apoe*^*−/−*^ or *Apoe*^*−/−*^*Nod1*^*−/−*^); ^#^*P* < 0.05; ^##^*P* < 0.01 *vs*. the corresponding sham condition. Bar size is 100 μm (panels A, B)

To determine the level of activation of the NOD1-dependent-pathway in the spleen from mice fed on HFD, splenic extracts were prepared from *Apoe*^*−/−*^, *Apoe*^*−/−*^*Nod1*^*−/−*^ and *Apoe*^*−/−*^ mice receiving intraperitoneally the NOD1-agonist iE-DAP (24 h before sacrifice) after 4 weeks of HFD. As Supplemental Figure S9D shows, in addition to RIPK2 phosphorylation, P-TAK1, a downstream target from P-RIPK2 also exhibited a minimal although statistically significant increase, which suggests that maximal NOD1 activation is not achieved only by feeding HFD. As expected, *Apoe*^*−/−*^*Nod1*^*−/−*^ mice failed to show this signaling. To evaluate the role of splenic NOD1 on atheroma dynamics, *Apoe*^*−/−*^ and *Apoe*^*−/−*^*Nod1*^*−/−*^ mice were submitted to sham operation or splenic artery ligation and fed on HFD for 4 weeks. As Fig. 3A shows, deletion of NOD1 reduced the atheromatous lesion in sham-operated mice, as previously described [31, 32, 35]. Interestingly, a significant reduction in the progression of the atheromatous lesion was also observed in *Apoe*^*−/−*^ animals that underwent spleen artery ligation, even lesser than that observed in sham-operated *Apoe*^*−/−*^*Nod1*^*−/−*^ mice. Importantly, *Nod1*^*−/−*^ mice fed on HFD for 4 weeks did not develop atherogenic lesions. However, and unexpectedly, splenic artery ligation in *Apoe*^*−/−*^*Nod1*^*−/−*^ mice abolished the decreased atheromatous lesion observed in the absence of NOD1 (Fig. 3A). These data indicate that the presence of the splenic remnant in the body provides signals that modulate atherogenesis, in addition to the effects dependent on NOD1 activation. Figure 3A (right panel) shows representative images of the lesion size.

Analysis of neutrophil infiltration and NETosis in the atheroma lesion was assessed in these histological sections, by quantifying the staining of Ly6G and citrullinated histone H3 (Fig. 3B). Additionally, the levels of CXCL12 in the lesion area were also quantified showing a significant decrease in the absence of NOD1 (Supplemental Figure S10). Moreover, splenic artery ligation reduced the levels of CXCL12 independently of the absence of NOD1 (Supplemental Fig. S10).

This protective role of NOD1 after splenic artery ligation was not associated with changes in the serum levels of triglycerides (TAG) or LDL; in fact, *Apoe*^*−/−*^*Nod1*^*−/−*^ mice exhibited higher levels of LDL regardless of spleen sham or artery ligation. Interestingly, ligation enhanced HDL and NEFA levels, which can be associated with the protective role of NOD1 under these conditions (Fig. 3C). Additionally, other humoral and cellular factors associated with spleen ligation need to be considered.

### The absence of NOD1 improves splenic lipid homeostasis and enhances neutrophil mobilization from the bone marrow

**Fig. 4:**
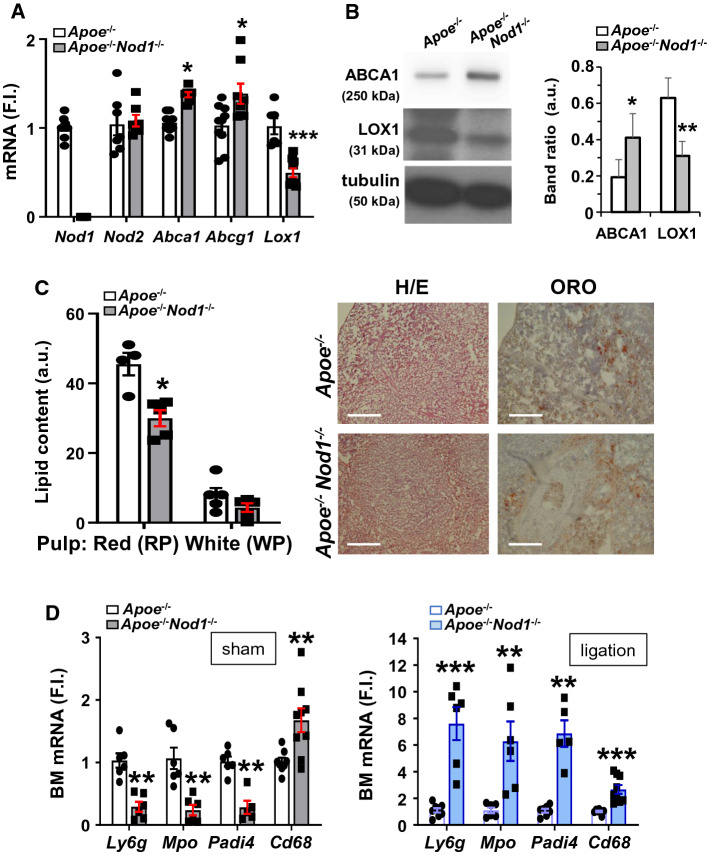
*Apoe*^*−/−*^*Nod1*^*−/−*^ mice fed on HFD have reduced lipid content in the spleen and diminished neutrophil-associated biomarkers in the bone marrow. **A** Splenic mRNA levels of *Nod1* and *Nod2* genes and the cholesterol and lipid efflux genes *Abca1* and *Abcg1*, and the oxLDL receptor *Lox1* in *Apoe*^*−/−*^ and *Apoe*^*−/−*^*Nod1*^*−/−*^ mice after 4 weeks of HFD. **B** Western blot analysis of the oxLDL receptor (LOX1) and the ABC transporter ABCA1 in samples from panel A. **C** Splenic lipid content in *Apoe*^*−/−*^ and *Apoe*^*−/−*^*Nod1*^*−/−*^ mice fed on HFD. **D** mRNA levels of genes associated with neutrophil content in the bone marrow from sham and spleen-ligated mice. Results show the mean ± SD from 7 animals of each condition (sham and ligation of *Apoe*^*−/−*^ and *Apoe*^*−/−*^*Nod1*^*−/−*^ mice). Statistical significance was estimated as *p* value calculated by un-paired *t* test; *P < 0.05; **P < 0.01; ***P < 0.005 vs. the corresponding *Apoe*^*−/−*^ condition. Bar size is 100 μm (panel C)

The absence of *Nod1* in HFD-fed *Apoe*^*−/−*^mice does not alter *Nod2* levels in the spleen but increases the expression of genes involved in the efflux of cholesterol (i.e., *Abca1* and *Abcg1*) at the time that decreases the expression of the oxLDL receptor *Lox1* (Fig. 4A). LOX1 protein levels decreased in splenic *Apoe*^*−/−*^*Nod1*^*−/−*^ mice, whereas those of the ABC transporter ABCA1, involved in the efflux of cholesterol, were increased (Fig. 4B). Accordingly, the lipid content, especially in the red pulp of the spleen, decreased in *Apoe*^*−/−*^*Nod1*^*−/−*^ mice *vs*. *Apoe*^*−/−*^ counterparts (Fig. 4C). Interestingly, the bone marrow from *Apoe*^*−/−*^ mice exhibited lesser content *vs. Apoe*^*−/−*^*Nod1*^*−/−*^ counterparts of cells expressing *Ly6g, Mpo and Padi4*, genes associated with neutrophil content and function [31, 32, 40–45]; however, this profile was completely reversed after spleen ligation, suggesting that splenic NOD1 has a significant role in bone marrow retention of neutrophils (Fig. 4D). Interestingly, the level of *Cd68*, encoding for a receptor associated with macrophage capture of LDL particles, was elevated in the absence of NOD1, regardless of the splenic function (sham or splenic artery ligation).

### Deletion of NOD1 alters the splenic composition and reduces the release of neutrophil extracellular traps (NETs)

**Fig. 5:**
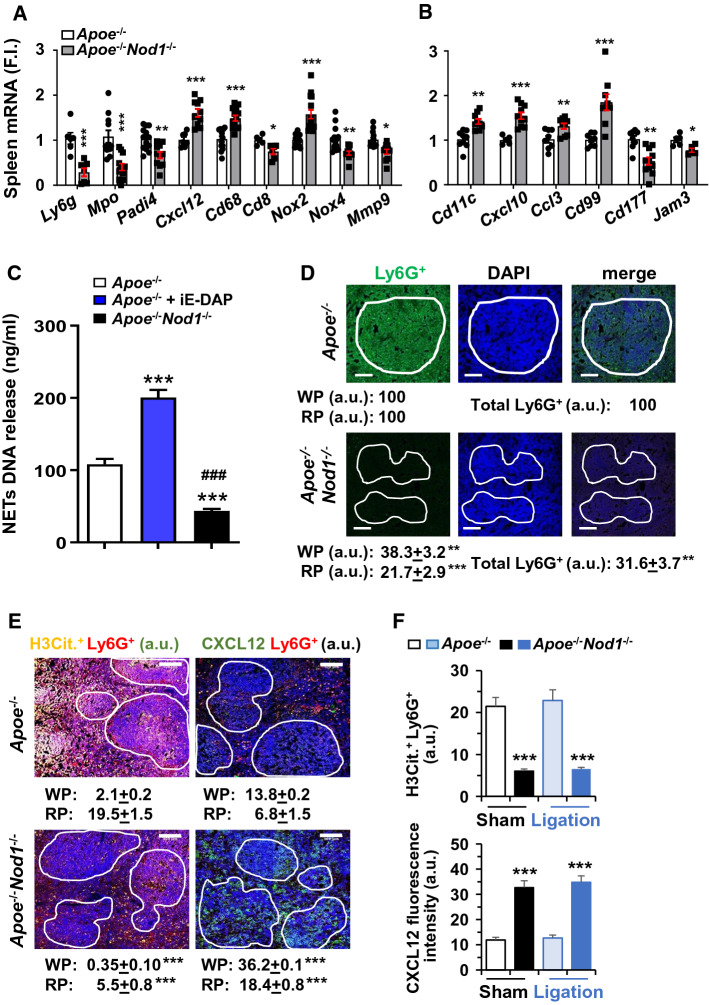
Deletion of NOD1 reduces neutrophil content and NETs release in the spleen from mice fed on HFD. **A**, **B** Splenic mRNA levels of genes related to neutrophil content in *Apoe*^*−/−*^ and *Apoe*^*−/−*^*Nod1*^*−/−*^ mice fed on HFD for 4 weeks. **C** DNA release (NETs) from neutrophils isolated from *Apoe*^*−/−*^ and *Apoe*^*−/−*^*Nod1*^*−/−*^ and *Apoe*^*−/−*^ mice challenged intraperitoneally with iE-DAP (1 mg/kg body weight) 24 h before sacrifice mice after 4 weeks of HFD. **D** Immunofluorescence analysis of Ly6G^+^ cells and DAPI staining in splenic sections from *Apoe*^*−/−*^ and *Apoe*^*−/−*^*Nod1*^*−/−*^ mice after 4 weeks of HFD. The white pulp (WP) is located within the indicated white line regions. The specific fluorescence signal located in the red pulp (RP) and the stromal tissue was quantified in the external region of the WP. **E**, **F** Quantification of citrullinated histone H3 associated with Ly6G^+^ cells (H3Cit.^+^ Ly6G^+^) and CXCL12 in spleen sections from *Apoe*^*−/−*^ and *Apoe*^*−/−*^*Nod1*^*−/−*^ mice (sham or after spleen artery ligation) fed 4 weeks HFD. Results show the mean ± SD from 8 animals of each condition (sham and artery ligation of *Apoe*^*−/−*^ and *Apoe*^*−/−*^*Nod1*^*−/−*^ mice). Statistical significance was estimated as P value calculated by un-paired *t-test*, or by one-way ANOVA followed by Bonferronis´s post hoc multi-comparisons analysis (panel C); *P < 0.05; **P < 0.01; ***P < 0.005 *vs*. the corresponding *Apoe*^*−/−*^ condition. ^###^*P* < 0.005 *vs*. the corresponding *Apoe*^*−/−*^ condition. Bar size is 50 μm (panels **D**–**F**)

One interesting feature of the spleen composition from *Apoe*^*−/−*^*Nod1*^*−/−*^ mice fed on HFD is the reduced presence of neutrophils as deduced by the decreased expression levels of the neutrophil markers *Ly6g* and *Mpo* (Fig. 5A). This is despite the increase in the serum levels of chemo-attractants (Fig. 2A). Indeed, other changes in gene transcription in the spleen related to NETs formation (Fig. 5A) and splenic trans-endothelial migration, such as *Cxcl12, Cxcl10, Cd177* and *Cd99* (Fig. 5B) were analyzed in *Apoe*^*−/−*^ and *Apoe*^*−/−*^*Nod1*^*−/−*^ mice after 4 weeks on HFD. Moreover, neutrophils isolated from *Apoe*^*−/−*^ mice fed on HFD exhibited a higher capacity to display the formation of NETs than those from the corresponding *Apoe*^*−/−*^*Nod1*^*−/−*^ mice. This capacity to release NETs by *Apoe*^*−/−−*^ mice was enhanced after NOD1 activation with iE-DAP (Fig. 5C). In addition, we quantified the levels of Ly6G^+^ splenic cells (Fig. 5D) that were significantly decreased in *Apoe*^*−/−*^*Nod1*^*−/−*^ mice. Since neutrophils produce NETs, leading to NETosis, the amounts of H3Cit. (marker of NETs [46, 47]) and the chemotactic CXCL12 were quantified in splenic sections from *Apoe*^*−/−*^ and *Apoe*^*−/−*^*Nod1*^*−/−*^ mice. As Fig. 5E shows, NETosis, determined by the H3Cit. content, was decreased in sham-operated *Apoe*^*−/−*^*Nod1*^*−/−*^* vs*. *Apoe*^*−/−*^ mice, whereas an increase in CXCL12 was observed in these sections, in agreement with the mRNA levels (Fig. 5A). Similar results were observed after spleen ligation (Fig. 5F).

**Fig. 6:**
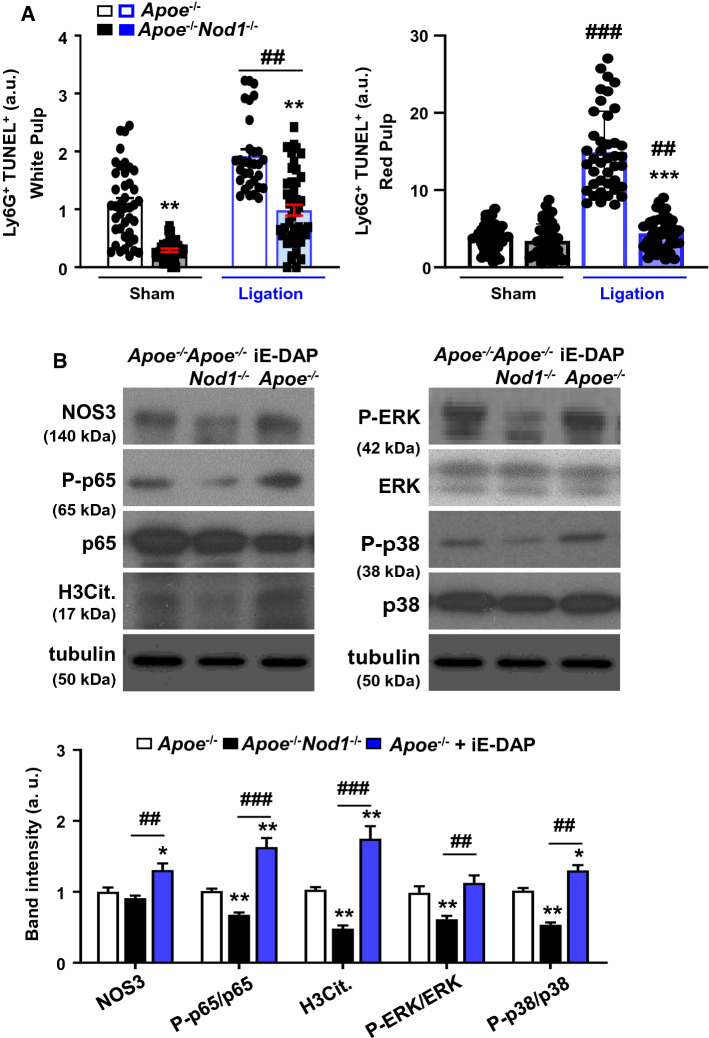
Spleen ligation reduces apoptosis and neutrophil splenic content in NOD1-deficient mice fed on HFD. Quantification of (**A**) TUNEL^+^ Ly6G^+^ cells in the splenic red pulp and white pulp from sham and spleen artery-ligated *Apoe*^*−/−*^ and *Apoe*^*−/−*^*Nod1*^*−/−*^ mice fed on HFD for 4 weeks. (**B**) Western blot analysis of proteins related to NETs and NETosis from *Apoe*^*−/−*^, *Apoe*^*−/−*^*Nod1*^*−/−*^ and *Apoe*^*−/−*^ mice challenged intraperitoneally with iE-DAP (1 mg/kg body weight) 24 h before sacrifice. Results show the mean ± SD from 7 animals of each condition (sham and ligation of *Apoe*^*−/−*^ and *Apoe*^*−/−*^*Nod1*^*−/−*^ mice). Statistical significance was estimated as P value calculated by un-paired *t-test* (panel A) o by one-way ANOVA followed by Bonferronis´s post hoc multi-comparisons analysis (Panel A, ligation vs. sham; B); *P < 0.05; **P < 0.01; ***p < 0.005 *vs*. the corresponding *Apoe*^*−/−*^ condition. ^##^*P* < 0.01; ^###^*P* < 0.005 *vs*. *Apoe*^*−/−*^*Nod1*^*−/−*^

Analysis of TUNEL^+^ cells in the splenic Ly6G^+^ population showed higher percentages in the white pulp of *Apoe*^*−/−*^* vs*. *Apoe*^*−/−*^*Nod1*^*−/−*^ mice regardless of splenic function (sham or after artery ligation; Fig. 6A). These differences were significantly enhanced in the red pulp of the spleens after artery ligation (Fig. 6A). To assess the extent of NETs formation under these conditions, *Apoe*^*−/−*^ and *Apoe*^*−/−*^ mice challenged with iE-DAP were compared to *Apoe*^*−/−*^*Nod1*^*−/−*^ mice. As Fig. 6B shows, endothelial nitric oxide synthase (NOS3) levels were increased in *Apoe*^*−/−*^ mice treated with iE-DAP, in agreement with the effect of NO on the regulation of atherogenesis and NETosis [48, 49]; however, the interplay of all of these issues with NOD1 activation has not been investigated before. Moreover, an increase in P-p65, P-ERK and P-p38 and mainly in H3Cit. content was evidenced in *Apoe*^*−/−*^ and *Apoe*^*−/−*^ treated with iE-DAP. These results suggest that, in the absence of NOD1, the citrullination of histone H3, as a marker of NETs formation was significantly undetected in the spleen. Also, the enhanced response observed after iE-DAP treatment suggests that NOD1 from splenic *Apoe*^*−/−*^ mice retained the capacity to fully express the maximal activity of this NOD1-dependent NETosis.

## Discussion

The role of the spleen in the aftermath of several diseases remains a conflictive issue due to its complexity in cell composition and cell-to-cell interactions [11, 38, 50], the presence of highly specialized microcompartments, and the continuous blood flow through this tissue [11, 51, 52]. Most of the physio-pathological performances of the spleen have been deduced after traumatic or therapeutic total or partial surgical removal of the organ. This is important since, in the USA for example, more than 20,000 surgical splenectomies per year are performed, and the side effects of these interventions are poorly studied [53]. Indeed, partial splenectomy remains a surgical option versus complete spleen removal to preserve organ function and avoid severe consequences resulting from the impairment of its normal metabolism [22, 54–57].

Here, we have investigated the role of the splenic NOD-like receptor NOD1 in both leukocyte trafficking and the subsequent progress of atherogenesis in mice fed on HFD, as previously observed [31, 32, 35]. First, we observed that splenic NOD1 was increased and was active in the spleen of *Apoe*^*−/−*^ mice fed on HFD, as reflected by the presence of downstream targets, such as phospho-RIPK2, phospho-TAK1 or phospho-p65 from the NF-κB pathway. This was probably due to the presence of oxidized LDL particles coming from the HFD/hypercholesterolemic diet and molecules derived from the microbiota (peptidoglycans) that are agonists of NOD1 [31, 32, 39, 58, 59]. This activation of the NOD1 pathway was near the range of the activity achieved after administration of the NOD1-agonist iE-DAP. Interestingly, NOD2 mRNA levels remained unchanged under these conditions. Moreover, even in the absence of NOD1, NOD2 levels did not change, pointing to a specific key role for NOD1 in the response of the spleen to HFD. However, this role of NOD1 seems to be paradoxical in terms of leukocytes mobilization from the bone marrow. Our data show that *Apoe*^*−/−*^*Nod1*^*−/−*^, compared to *Apoe*^*−/−*^ mice, exhibit an enhanced presence of chemoattractant chemokines in the serum of animals fed on HFD, in particular CCL2, CXCL1 [60] and CXCL2 [61, 62]. Under this context, a flow of CD45^+^ cells from the bone marrow to the systemic circulation, but not to the spleen, occurs. Among them, immature myeloid cells (Ly6C^+^CD11b^+^) appear to exit the bone marrow and have been previously characterized [63, 64]. We hypothesized that part of these cells is mobilized toward the spleen due to the enhanced presence of CXCL12 in this organ [38]. In addition, this seems to be a direct effect of the HFD since the same type of mobilizations was observed in *Apoe*^*−/−*^ mice. These data fit with previous work using an alternative model of mice atherogenesis (*Ldlr*^*−/−*^) fed on HFD [60]. These mice also exhibit enhanced levels of circulating chemo-attractants and accumulation of neutrophils in the spleen with citrullinated histone H3. Furthermore, diets rich in saturated fatty acids enhance the depletion of cells from the bone marrow, whereas diets rich in polyunsaturated fatty acids contribute to the retention of neutrophils in the bone marrow, in line with our data [65].

**Fig. 7:**
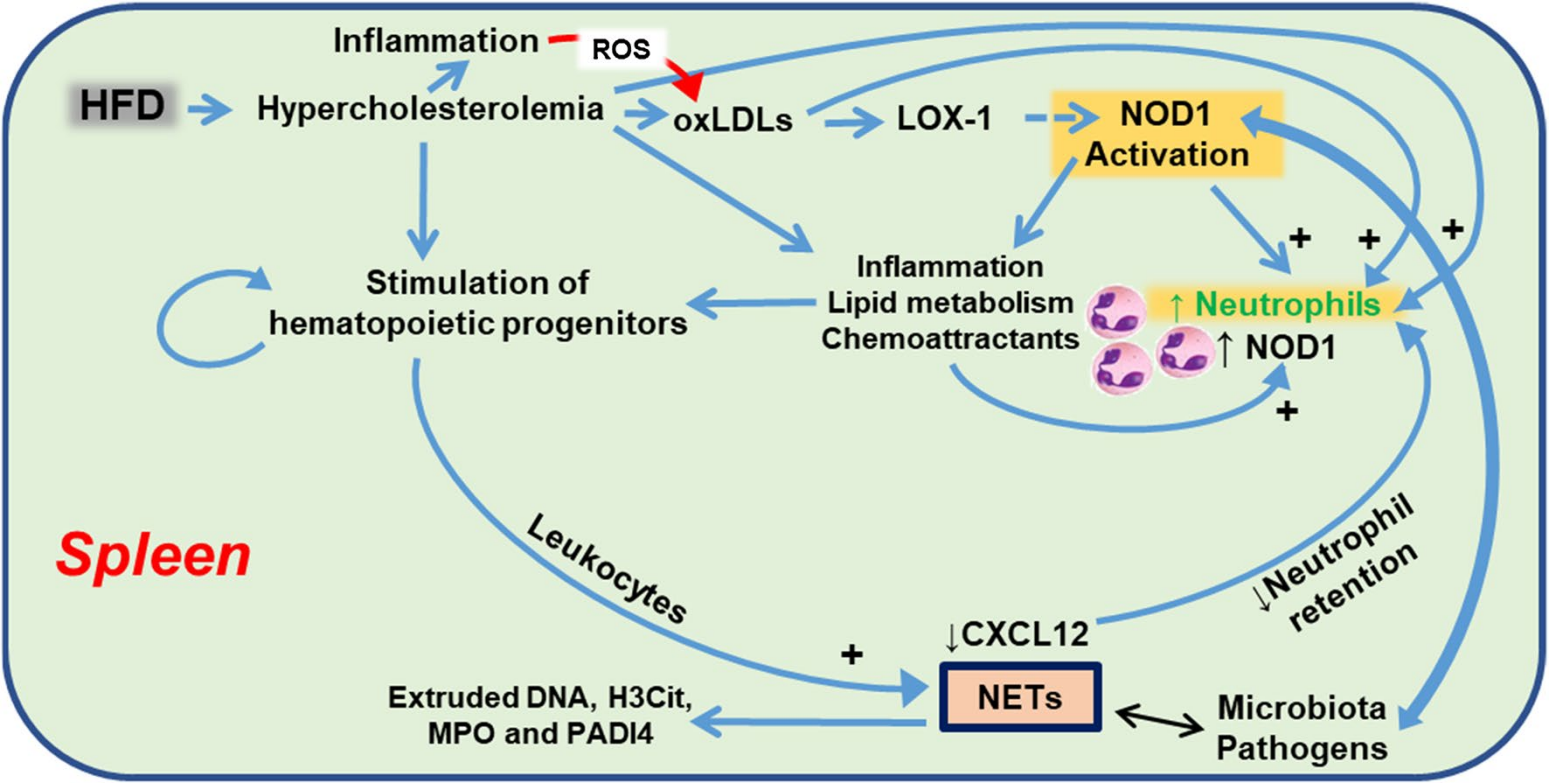
Schematic representation of the contribution of the splenic activity of NOD1 and HFD to the mobilization of immune cells. Under HFD conditions, NOD1 is activated in different cell types, including bone marrow and splenic cells. This activation promotes the ontogeny of hematopoietic precursors in the bone marrow. Under HFD, deficiency in *Nod1* reduces the pool of cells in the bone-marrow, promoting their accumulation in the blood. However, *Apoe*^*−/−*^*Nod1*^*−/−*^ mice fed on HFD fail to accumulate leukocytes in the spleen, especially neutrophils. In addition, activation of NOD1 in splenic neutrophils from *Apoe*^*−/−*^ mice exhibit citrullination of histone H3 and NETosis upon HFD feeding

HFD-fed *Apoe*^*−/−*^ mice are a classical model for induction of atherogenesis [66]. Here, we show that the fine-tuning of atherogenesis progression was also modulated by the spleen itself since splenic artery ligation leads to a significant reduction in the atherogenic lesion extent. One possibility to explain these results is the increase of NETs and TUNEL^+^/Ly6G^+^ cells in *Apoe*^*−/−*^ mice after spleen ligation. However, whereas we confirmed that *Apoe*^*−/−*^*Nod1*^*−/−*^ mice exhibit a reduced atheromatous lesion due to reduced recruitment of circulating inflammatory cells [32, 35], splenic artery ligation failed to support this protection, reflecting the involvement of different mechanisms in atherogenesis progression. Moreover, analysis of the lipidic profile in these animals showed minimal, but statistically significant, differences between *Apoe*^*−/−*^ and *Apoe*^*−/−*^*Nod1*^*−/−*^ mice in LDL levels and increased HDL levels after splenic artery ligation. Overall, *Apoe*^*−/−*^*Nod1*^*−/−*^ mice showed a decreased lipid content in the spleen, which implies that splenic NOD1 activation was involved in lipid regulation and accumulation in this organ. In this regard, the contribution of the spleen to atherosclerotic disease has been stressed by various groups [4, 5, 18, 67, 68]. A summary of the role of the spleen in the context of *Nod1* and *Apoe* deficiency and the signaling involved in the atherogenic progression is shown in Fig. 7.

Finally, our results support the view that both the spleen and the activation of NOD1 in splenic cells have a significant impact on the progress of atherogenesis under HFD. Furthermore, these results open the way for the design of novel therapeutic strategies based on NOD1 inhibition under conditions of plaque progression and potential atherothrombotic events.

## Concluding remarks

The involvement of the NLRs, and in particular of NOD1, in many inflammatory diseases is a growing field that links and stresses the importance of the diet and the metabolic changes derived from it in both the host microbiota and the immune system. This is because NOD1 agonists from the gut microbiota can access systemic circulation, playing a relevant function in gastrointestinal immune-metabolic adaptations. Here, we describe the role of splenic NOD1 activation in the outcome of the diet-induced atherogenesis and mobilization of different leukocyte populations from the bone marrow to vascular lesions. In addition, our data show that NOD1 deletion in mice fed on a chow diet did not alter the hematopoietic flow from the bone marrow to the blood and the spleen. However, *Nod1*^*−/−*^ mice fed on a high-fat diet (HFD) exhibit a significant mobilization of immune cells from the bone marrow into the circulation, accumulating in the spleen. Furthermore, under pro-atherogenic conditions due to HFD in *Apoe*^*−/−*^ mice, deletion of NOD1 has a significant impact on the accumulation of splenic myeloid cell subpopulations, an effect that is associated with changes in the circulating levels of chemoattractant factors. This increase in circulating immune cells in *Apoe*^*−/−*^*Nod1*^*−/−*^ mice, but its restricted infiltration in tissues with an inflammatory tendency (due to *Nod1* deficiency), contributes to reducing the atherogenic lesion. Interestingly, splenic artery ligation in *Apoe*^*−/−*^ mice-fed HFD reduces atherosclerotic disease progression, an effect that is lost in *Apoe*^*−/−*^*Nod1*^*−/−*^ mice. Finally, NOD1 activation under HFD conditions contributes to NETs formation and NETosis in the spleen, playing a role in splenic cells homeostasis and in the atheroma layer, further contributing to the development of cardiovascular diseases. The exact role of these NETs formation is unclear, but it can help to provide additional clues to understanding the mechanisms that contribute to the regulation of innate immunity and the potential adverse effects in the progression of low-grade pro-inflammatory diseases.

### Supplementary Information

Below is the link to the electronic supplementary material.

Supplementary file1 (PDF 1983 kb)

## Data Availability

The data generated and analyzed during the current study are available upon request to the authors.
