# Supplementary material for: High-fat diet activates splenic NOD1 and enhances neutrophil recruitment and neutrophil extracellular traps release in the spleen of ApoE-deficient mice
Source: Cell Mol Life Sci. 2022 Jul 5;79(8):396. doi: 10.1007/s00018-022-04415-x (PMC9256580; doi:10.1007/s00018-022-04415-x)
Supplement: Supplementary file 1 — Supplementary file1 (PDF 1983 kb) [file 18_2022_4415_MOESM1_ESM.pdf]

Supplementary material: Figures and Table

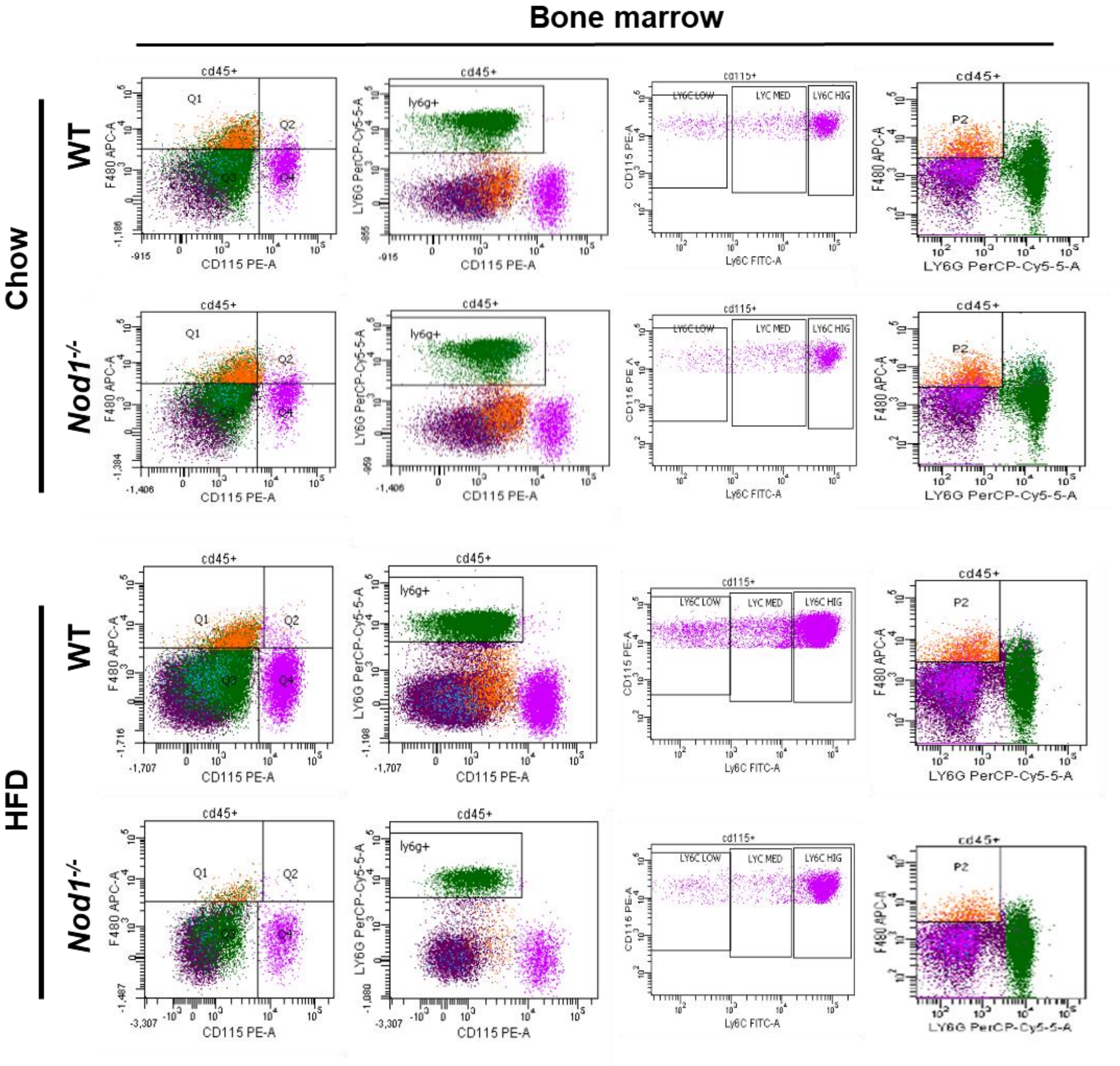

Figure S1. Flow cytometry analysis of the distribution and quantification of cell populations from the bone marrow of wild-type (WT) and *Nod1*$^{-/-}$ male mice fed chow or HFD. The plots correspond to the values indicated in Figure 1A. Results show a representative flow cytometry analysis.

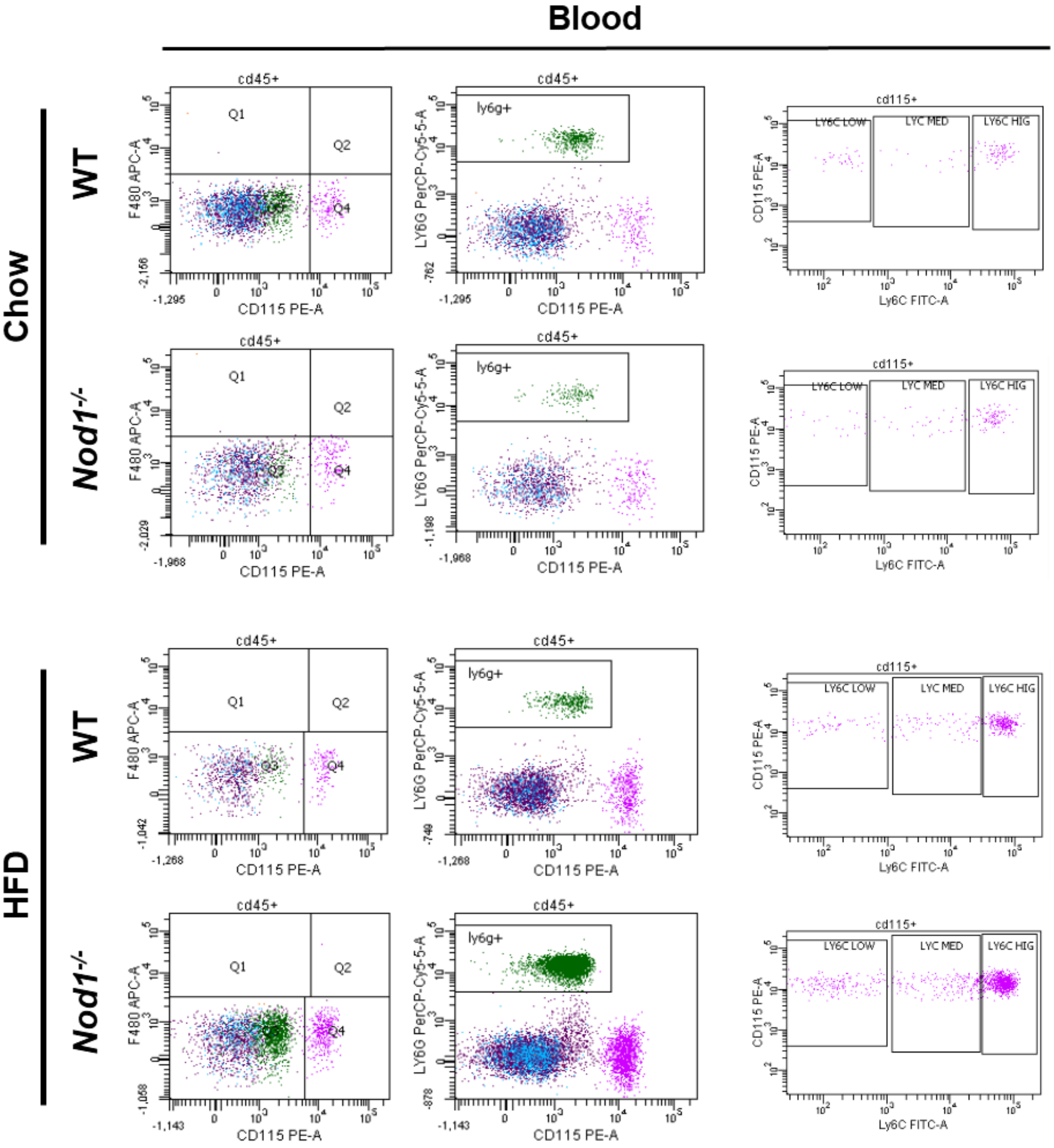

Figure S2. Flow cytometry analysis of the distribution and quantification of cell populations from the blood of wild-type (WT) and *Nod1*$^{-/-}$ male mice fed chow or HFD. The plots correspond to the values indicated in Figure 1B. Results show a representative flow cytometry analysis.

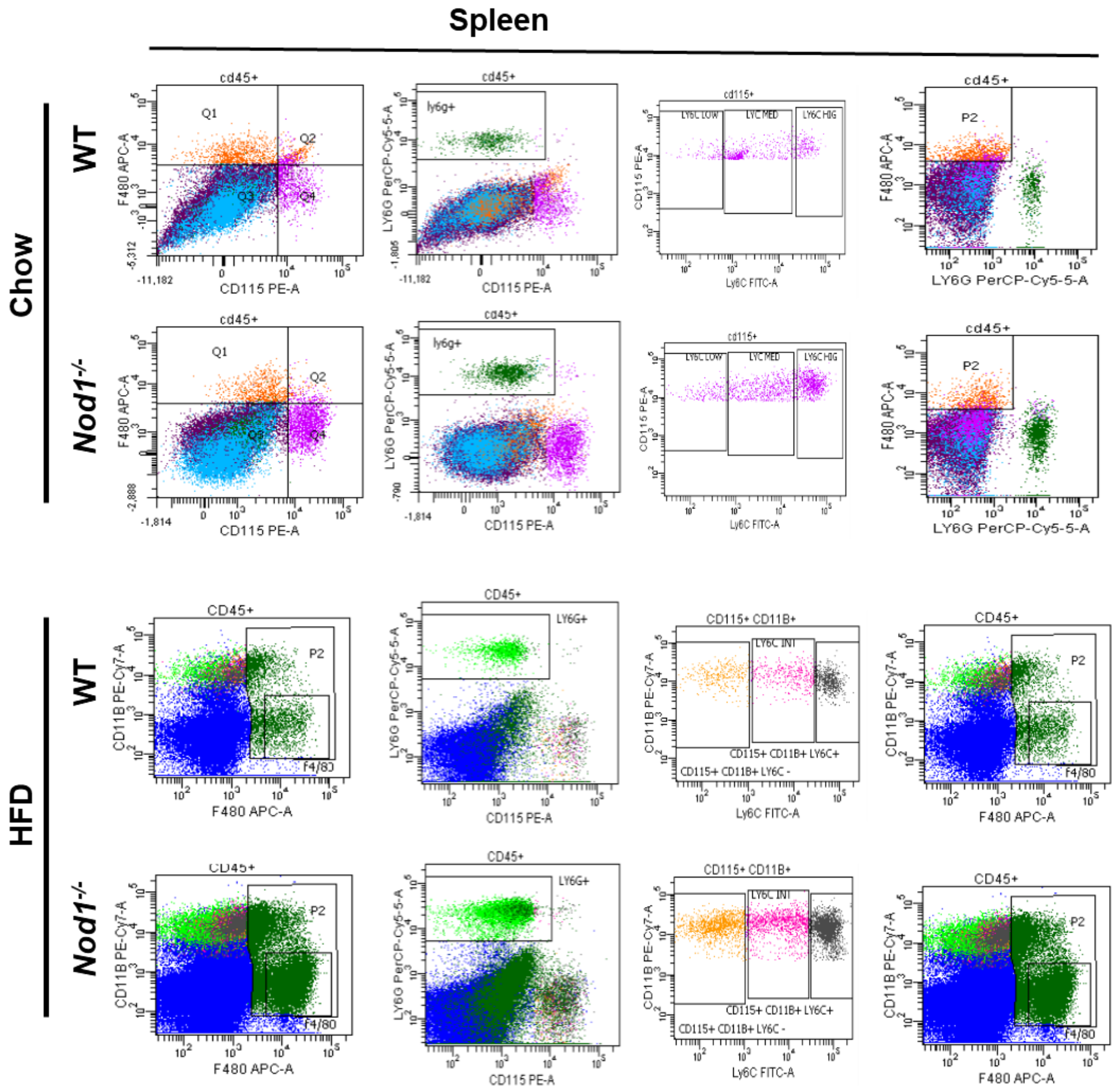

Figure S3. Flow cytometry analysis of the distribution and quantification of cell populations from the spleen of wild-type (WT) and *Nod1*$^{-/-}$ male mice fed chow or HFD. The plots correspond to the values indicated in Figure 1C. Results show a representative flow cytometry analysis.

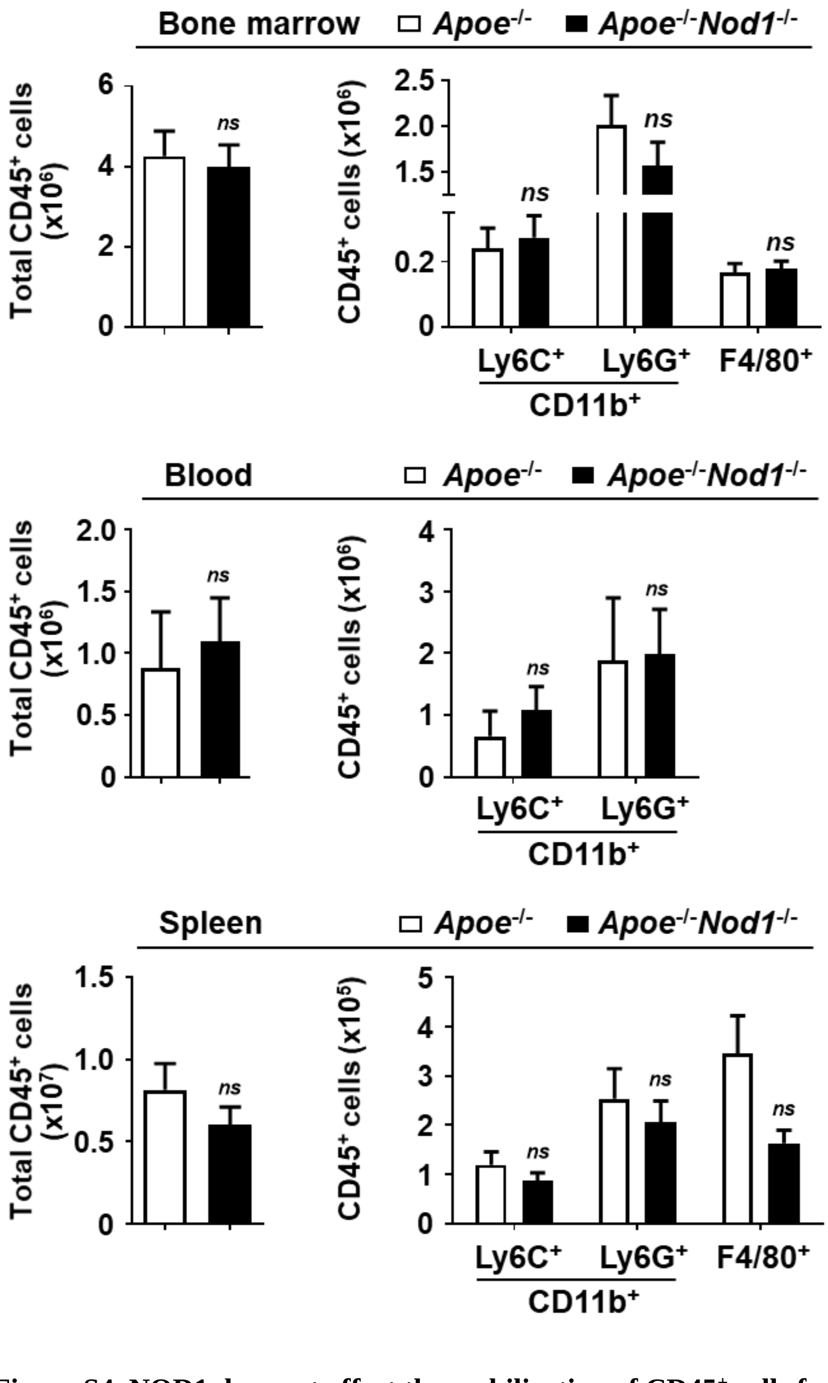

Figure S4. NOD1 does not affect the mobilization of $CD45^+$ cells from the bone marrow in mice fed chow diet. *Apoe*$^{-/-}$ and *Apoe*$^{-/-}$*Nod1*$^{-/-}$ male mice were fed chow diet for the same period as those fed HFD (Figures 1 to 6). The amount and distribution of $CD45^+$ cells were determined in the bone marrow, blood and spleen. The content of $CD45^+$ cells and the $CD11b^+Ly6C^+$, $CD11b^+Ly6G^+$ and $F4/80^+$populations were determined. Results show the mean $\pm$ SD from 6 animals of each condition. *ns*: not statistically significant vs. the corresponding *Apoe*$^{-/-}$ mice.

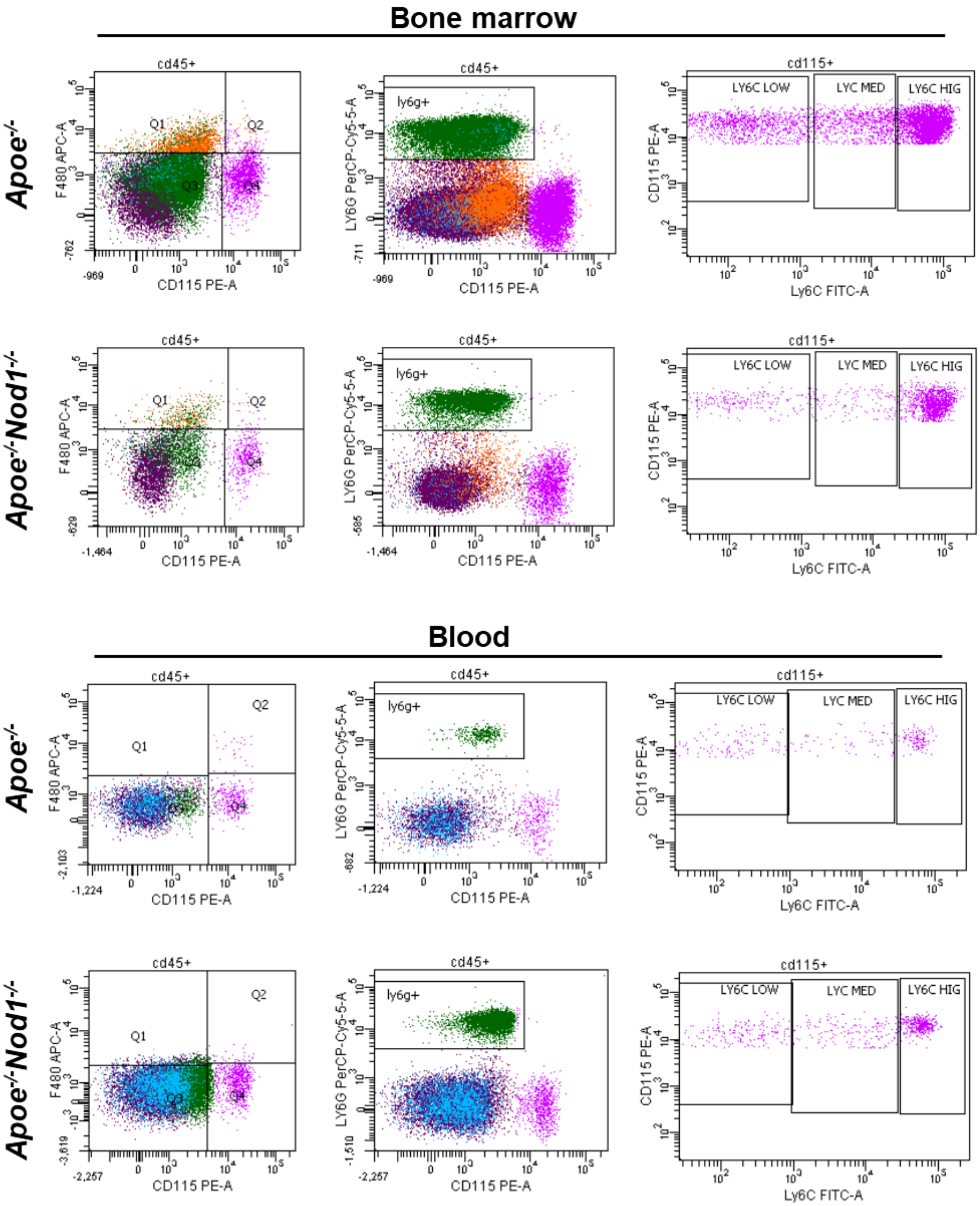

Figure S5. Flow cytometry analysis of the distribution and quantification of cell populations from the bone marrow and the blood of *Apoe*$^{-/-}$ and *Apoe*$^{-/-}$*Nod1*$^{-/-}$ male mice fed HFD for four weeks. The plots correspond to the values indicated in Figure 2B. Results show a representative flow cytometry analysis.

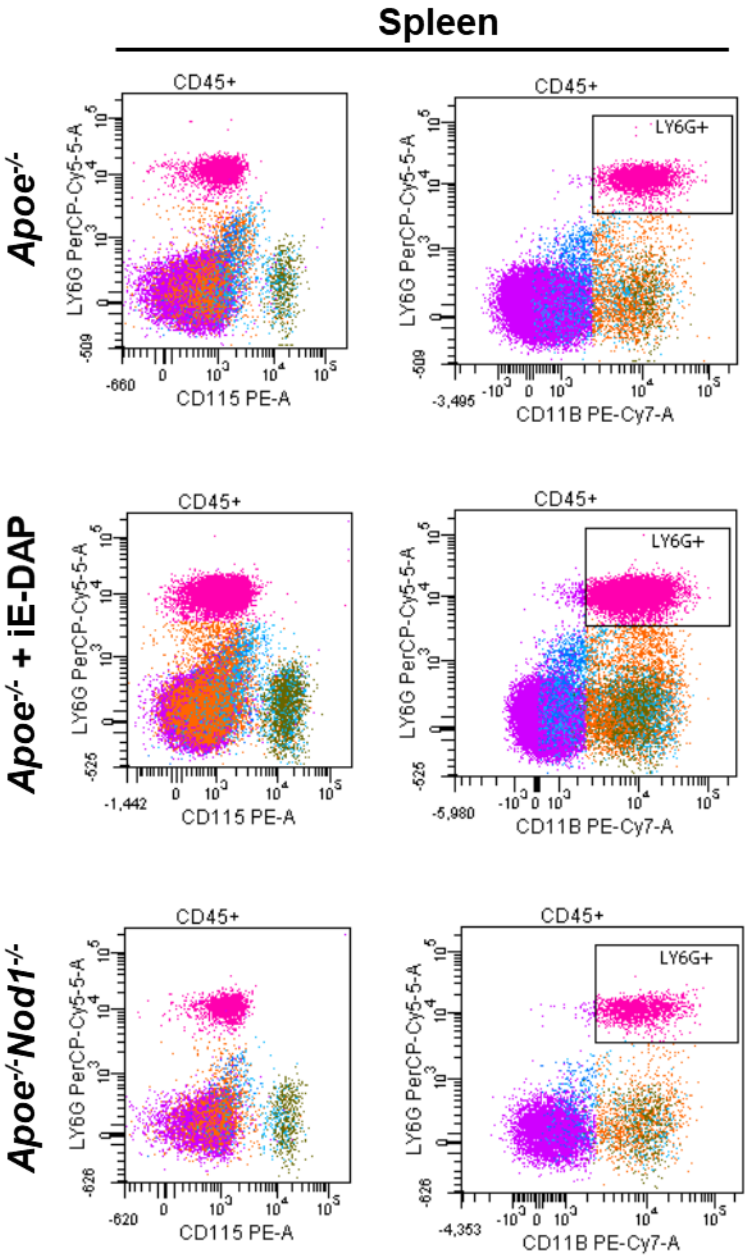

Figure S6. Flow cytometry analysis of the distribution and quantification of cell populations from the spleen of *Apoe*$^{-/-}$ and *Apoe*$^{-/-}$*Nod1*$^{-/-}$ male mice fed HFD for four weeks. The plots correspond to the values indicated in Figure 2C. The NOD1 activator iE-DAP (1 mg/Kg body weight) was administered to *Apoe*$^{-/-}$ at the end of the HFD treatment, 24h before sacrifice. Results show a representative flow cytometry analysis.

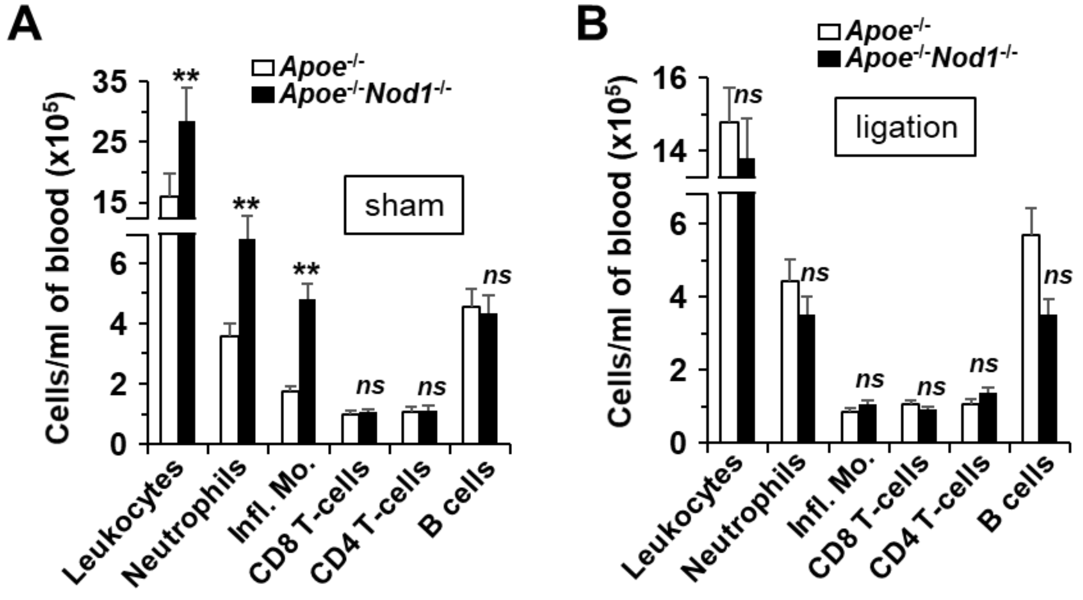

Figure S7. Contribution of NOD1 to blood immune cell profile under pro-atherogenic conditions and role of splenic function. *Apoe$^{-/-}$* and *Apoe$^{-/-}$Nod1$^{-/-}$* mice were submitted to sham or splenic artery-ligation followed by 4 weeks of HFD. The indicated cell populations were determined by flow cytometry as described in the methods section. (A) Circulating immune cells from mice after sham, (B) spleen artery-ligation. Values were expressed as total cell counts. Results show the mean $\pm$ SD from 8 animals of each condition (sham and ligation of *Apoe$^{-/-}$* and *Apoe$^{-/-}$Nod1$^{-/-}$*). Statistical significance was estimated as P value calculated by un-paired *t-test*; *P<0.05; **P<0.01; ***P<0.005 *vs*. the corresponding splenic intervention condition (*Apoe$^{-/-}$* or *Apoe$^{-/-}$Nod1*). *ns*: not statistically significant vs. the corresponding *Apoe$^{-/-}$* mice condition.

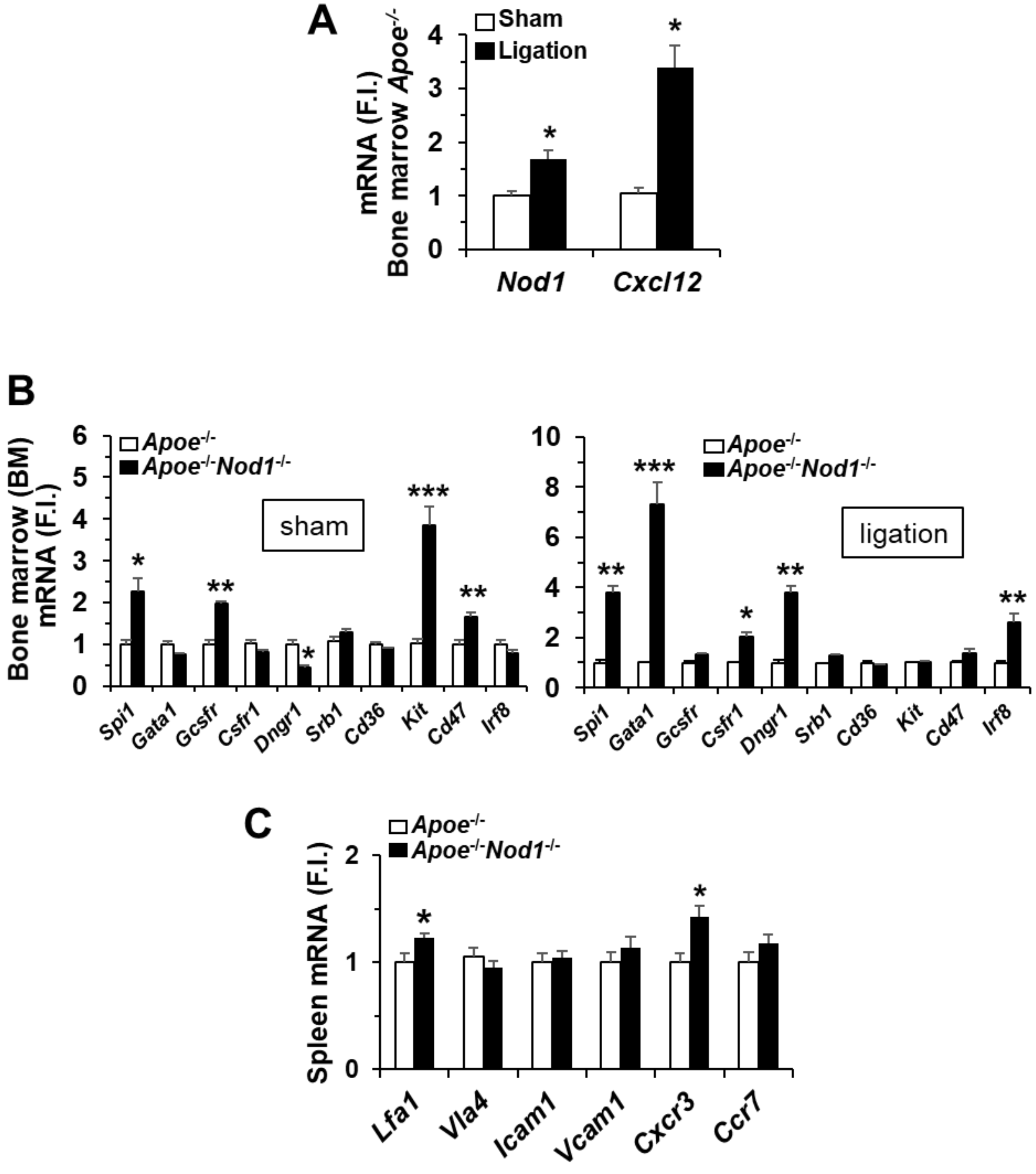

Figure S8. Splenic loss-of-function alters NOD1 expression and cell fate in the bone marrow. *Apoe*$^{-/-}$ and *Apoe*$^{-/-}$*Nod1*$^{-/-}$ mice were submitted to sham or splenic artery-ligation followed by 4 weeks of HFD. (A) Effect of spleen ligation on bone marrow mRNA levels of *Nod1* and *Cxcl12* genes in *Apoe*$^{-/-}$ mice fed HFD. (B) Contribution of NOD1 to specific expression of lineage commitment genes in the bone marrow after sham or splenic-artery ligation. (C) Analysis of mRNA expression of splenic genes involved in chemoattractant activity in *Apoe*$^{-/-}$ and *Apoe*$^{-/-}$*Nod1*$^{-/-}$ mice fed HFD. Results show the mean $\pm$ SD from 7 animals of each condition (sham and ligation of *Apoe*$^{-/-}$ and *Apoe*$^{-/-}$*Nod1*$^{-/-}$ mice). Statistical significance was estimated as P value calculated by un-paired *t-test*; *P<0.05; **P<0.01; ***P<0.005 *vs*. the corresponding sham (A) or *Apoe*$^{-/-}$ condition (B-C).

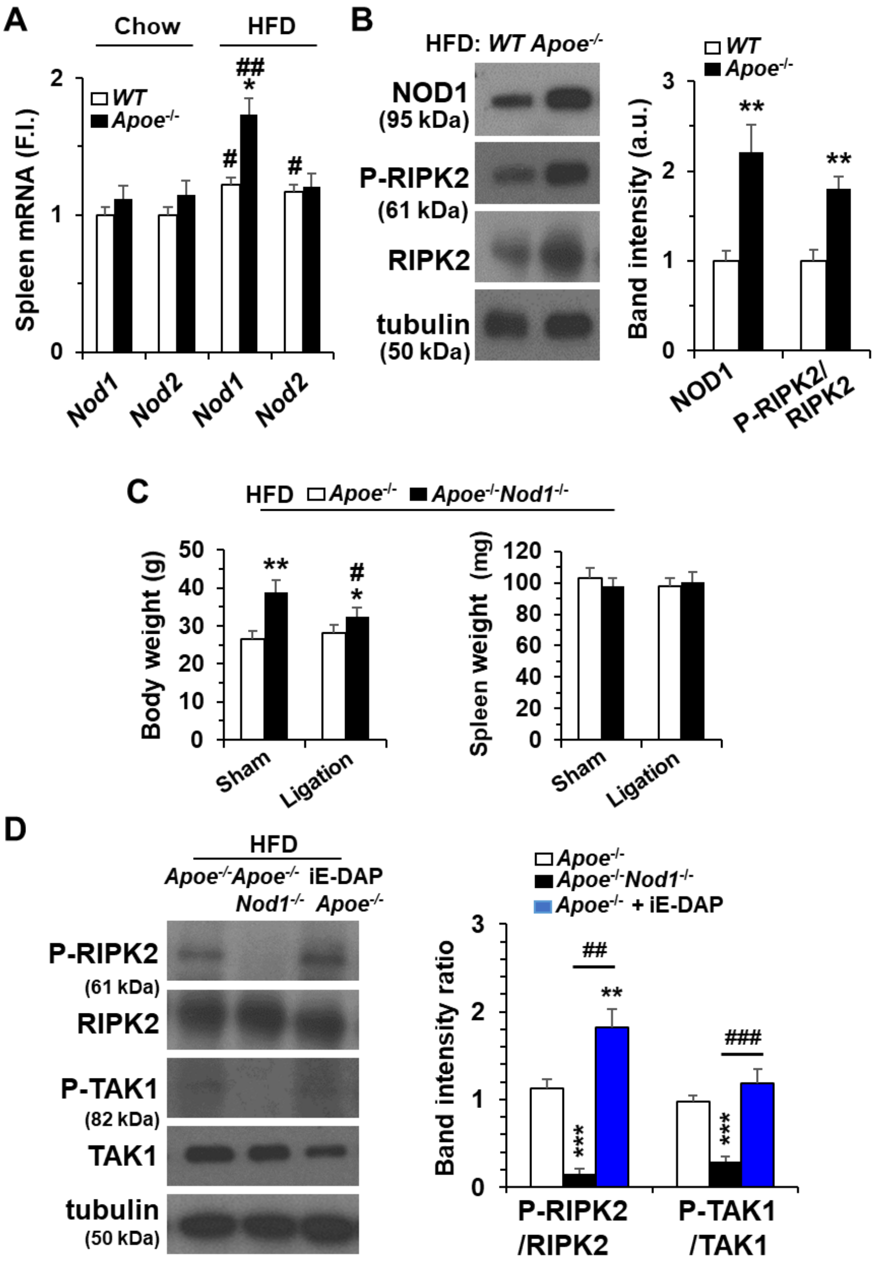

Figure S9. Splenic NOD1 is activated in *Apoe*$^{-/-}$ mice fed HFD. (A) *Nod1* and *Nod2* mRNA levels in WT and *Apoe*$^{-/-}$ mice fed chow or HFD for 4 weeks, and referred to the WT chow condition. (B) NOD1 activation pathway from mice described in panel A. (C) Body weight and spleen weight of *Apoe*$^{-/-}$ and *Apoe*$^{-/-}$*Nod1*$^{-/-}$ mice fed HFD for four weeks. (D) The NOD1 activator iE-DAP (1 mg/Kg body weight) was administered to *Apoe*$^{-/-}$ at the end of the HFD treatment, 24h before sacrifice. Spleen extracts were prepared and the levels of P-RIPK2, RIPK2, P-TAK1, TAK1 and tubulin were determined by Western blot and quantified. Results show the mean ± SD from 9 animals of each condition (WT, *Apoe*$^{-/-}$ and *Apoe*$^{-/-}$*Nod1*$^{-/-}$), or a representative blot (B,D) out of four. Statistical significance was estimated as P value calculated by un-paired t-test or by one-way ANOVA followed by Bonferronis´s *post hoc* multicomparisons analysis (panel C and D); *P<0.05; **P<0.01; ***P<0.005 *vs*. the corresponding control condition (WT or *Apoe*$^{-/-}$); $^{\#}$P<0.05; $^{\#\#}$P<0.01; $^{\#\#\#}$P<0.005 *vs*. chow condition (A) or *Apoe*$^{-/-}$*Nod1*$^{-/-}$ mice (B-D).

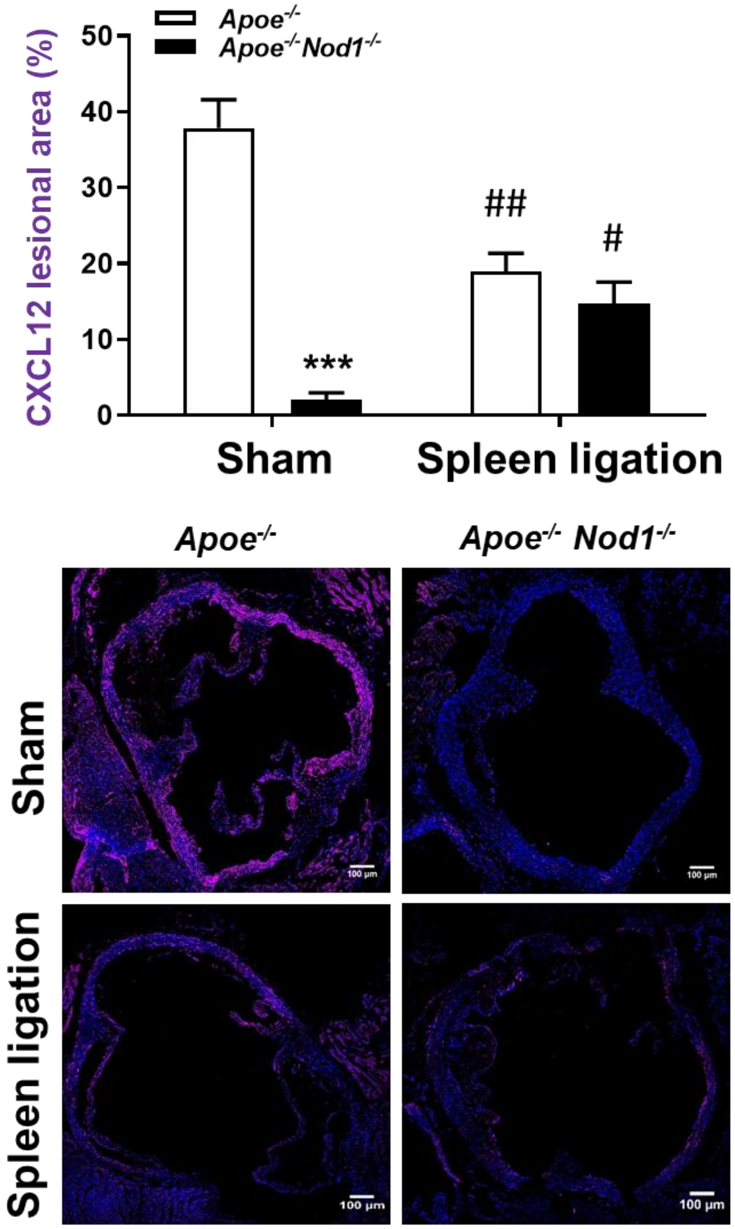

Figure S10. The CXCL12 content in atherogenic lesions in *Apoe*$^{-/-}$ mice fed HFD is reduced in the absence of NOD1 or after splenic artery-ligation. *Apoe*$^{-/-}$ and *Apoe*$^{-/-}$*Nod1*$^{-/-}$ mice were submitted to sham or splenic artery-ligation before start a 4 weeks HFD. The presence of CXCL12 in the atherogenic lesion was quantified by histochemistry (*upper panel*). Results show the mean $\pm$ SD from 6 animals of each condition (sham and splenic artery-ligation of *Apoe*$^{-/-}$ and *Apoe*$^{-/-}$*Nod1*$^{-/-}$ mice). Statistical significance was estimated as P value calculated by un-paired t-test and by one-way ANOVA followed by Bonferronis´s *post hoc* multicomparisons analysis; ***P<0.005 *vs*. the corresponding *Apoe*$^{-/-}$ condition; $^{\#}$P<0.05; $^{\#\#}$P<0.01 *vs*. the corresponding sham condition (*Apoe*$^{-/-}$ or *Apoe*$^{-/-}$*Nod1*$^{-/-}$). Representative immunohistochemistry images (*lower panel*). Bar size is 100 µm.

Supplemental Table S1. Oligonucleotide sequences designed for RT-PCR analysis

GENE	FORWARD SEQUENCE	REVERSE SEQUENCE
Abca1	ATAGCAGGCTCCAACCCTGAC	GGTACTGAAGCATGTTTCGATGTT
Abcg1	CTTTCCTACTCTGTACCCGAGG	CGGGGCATTCCATTGATAAGG
Ccl3	TGTACCATGACACTCTGCAAC	CAACGATGAATTGGCGTGGAA
Ccr7	TGTACGAGTCGGTGTGCTTC	GGTAGGTATCCGTCATGGTCTTG
Cd11c	CCAAGACATCGTGTTCCTGATT	ACAGCTTTAACAAAGTCCAGCA
Cd177	ATACCAGTGCTGACCCTTCTG	CCTCGCAGGTTTTCTCACCA
Cd36	AGATGACGTGGCAAAGAACAG	CCTTGGCTAGATAACGAACTCTG
Cd47	TGGTGGGAAACTACACTTGCG	CGTGCGGTTTTTCAGCTCTAT
Cd68	TGTCTGATCTTGCTAGGACCG	GAGAGTAACGGCCTTTTTGTGA
Cd8	CTCTGCCCTCATTCAGACCC	AGATGCTTTTAACCTCACAGGAC
Cd99	GCCTCGCCTGAATATGCAAA	GTCAGTTGTGGGCGGAGTCTT
Csfr1	TGTCATCGAGCCTAGTGGC	GGTCCAAGGTCCAGTAGGG
Cxcl10	CCAAGTGCTGCCGTCATTTTC	GGCTCGCAGGGATGATTTCAA
Cxcl12	TGAGCTACAGATGCCCATGC	TTCTCCAGGTACTCCTGAATCC
Cxcr3	GGTTAGTGAACGTCAAGTGCT	CCCCATAATCGTAGGGAGAGGT
Dngr1	GAAGTGCCAATCCCCTAGCAA	CAGTCACTACCTGAATGGAGAGA
Jam3	CTGCGACTTCGACTGTACG	TTCGGTTGCTGGATTTGAGATT
Gata1	TATGGCAAGACGGCACTCTAC	GGTGTCCAAGAACGTGTTGTT
Gcsfr1	CTGATCTTCTTGCTACTCCCCA	GGTGTAGTTCAAGTGAGGCAG
Icam1	GTGATGCTCAGGTATCCATCCA	CACAGTTCTCAAAGCACAGCG
Irf8	CGGGGCTGATCTGGGAAAAT	CACAGCGTAACCTCGTCTTC
Kit	GGCCTCACGAGTTCTATTTACG	GGGGAGAGATTTCCCATCACAC
Lfa1	AGAAGCCACCATTTCCCTCT	TGCTTGTTCGGCAGTGATAG
Lox1	CAAGATGAAGCCTGCGAATGA	ACCTGGCGTAATTGTGTCCAC
Ly6g	TGGACTCTCACAGAAGCAAAG	GCAGAGGTCTTCCTTCCAACA
Mmp9	GGACCCGAAGCGGACATTG	CGTCGTCGAAATGGGCATCT
Mpo	AGTTGTGCTGAGCTGTATGGA	CGGCTGCTTGAAGTAAAACAGG
Nod1	CCCCTTCCCAGCTCATTCG	GTGTCCATATAGGTCTCCTCCA
Nod2	TGGACACAGTCTGGAACAAGG	CAGGACCCATACAGTTCAAAGG
Nox2	TGTGGTTGGGGCTGAATGTC	CTGAGAAAGGAGAGCAGATTTCG
Nox4	CCTTTTACCTATGTGCCGGAC	CATGTGATGTGTAGAGTCTTGCT
Padi4	CAAGACAGTGTGACGTTCCGT	GGCCTGGATGTAGCCAATCT
Spi1	TTACAGGCGTGCAAAATGGAA	GACGTTGGTATAGCTCTGAATCG
Srb1	CGAAGTGGTCAACCCAAACGA	CCATGCGACTTGTCAGGCT
Vcam1	AGTTGGGGATTCGGTTGTTCT	CCCCTCATTCCTTACCACCC
Vla4	CACTCCAGCCGATCCTTCA	TGCAGGCAAGCTTCACTATG
